# Lifetime risk of severe kidney disease in lithium-treated patients: a retrospective study

**DOI:** 10.1186/s40345-023-00319-2

**Published:** 2023-12-09

**Authors:** Mihaela Golic, Harald Aiff, Per-Ola Attman, Bernd Ramsauer, Staffan Schön, Steinn Steingrimsson, Jan Svedlund

**Affiliations:** 1 Department of Psychiatry and Neurochemistry, Institute of Neuroscience and Physiology, Sahlgrenska Academy, University of Gothenburg, Gothenburg, Sweden; 2 Department of Psychiatry, Region Halland, Varberg, Sweden; 3 Psykiatri Affektiva, Department of Psychiatry, Region Västra Götaland, Gothenburg, Sweden; 4 Department of Nephrology, Institute of Medicine, Sahlgrenska Academy, University of Gothenburg, Gothenburg, Sweden; 5 Department of Nephrology, Skaraborg Hospital, Skövde, Sweden; 6 Swedish Renal Registry, Jönköping County Hospital, Jönköping, Sweden

**Keywords:** Lithium, Side effects, Chronic kidney disease, Lithium nephropathy, Renal impairment, Time on lithium, Treatment duration, Lithium exposure, Long-term

## Abstract

**Background:**

Lithium is an essential psychopharmaceutical, yet side effects and concerns about severe renal function impairment limit its usage.

**Aims:**

Our objectives were to quantify the occurrence of chronic kidney disease stage 4 or higher (CKD4 +) within a lithium-treated population, using age- and time-specific cumulative incidence and age-specific lifetime risk as measures of disease occurrence. Additionally, we aimed to investigate the association between the duration of lithium treatment and the risk of CKD4 + .

**Methods:**

We identified patients from the Sahlgrenska University Hospital’s laboratory database. We conducted a retrospective cohort study employing cumulative incidence functions that account for competing deaths to estimate cumulative and lifetime risk of CKD4 + . A subdistribution hazards model was employed to explore baseline covariates. For measuring the association between the duration of lithium treatment and CKD4 + occurrence, we used a matched 1:4 case–control study design and logistic regression.

**Results:**

Considering a 90-year lifetime horizon, the lifetime risk of CKD4 + for patients initiating lithium treatment between ages 55 and 74 ranged from 13.9% to 18.6%. In contrast, the oldest patient group, those starting lithium at 75 years or older, had a lower lifetime risk of 5.4%. The 10-year cumulative risk for patients starting lithium between ages 18 and 54 was minimal, ranging from 0% to 0.7%. Pre-treatment creatinine level was a predictive factor, with a hazard ratio of 4.6 (95% CI 2.75–7.68) for values within the upper third of the reference range compared to the lower third. Moreover, twenty or more years of lithium exposure showed a strong association with an increased risk of CKD4 + compared to 1–5 years of lithium use, with an odds ratio of 6.14 (95% CI 2.65–14.26).

**Conclusions:**

The risk of CKD4 + among lithium-treated patients exhibited significant age-related differences. Patients under 55 years old had negligible 10-year risk, while the lifetime risk for those aged 75 and older was limited. Duration of lithium treatment, especially exceeding 20 years, emerged as a significant risk factor. For individual risk assessment and prediction, consideration of age, pre-treatment creatinine levels, and the chosen time horizon for prediction is essential.

**Supplementary Information:**

The online version contains supplementary material available at 10.1186/s40345-023-00319-2.

## Background

Lithium, an established treatment for affective disorders, plays a pivotal role in managing bipolar disorder type I and offers a valuable option for bipolar disorder type II, as well as treatment-resistant unipolar depression, despite global variations in prescribing practices.

However, the use of lithium may be limited by side effects, most of which are clinically or laboratory observable and can occur at any point during the treatment. These side effects include: gastrointestinal reactions, tremor, polyuria, skin and hair conditions or endocrine disorders. Thyroid disorders can develop after a variable time interval, ranging from less than one to more than 25 years, and approximately one-third of patients will develop hypothyroidism (Joseph et al. 2023). The negative effect of lithium on kidney function develops slowly, is asymptomatic for years and has been a subject of ongoing discussion.

Although research outcomes over the past decade have not entirely aligned, there is a general consensus that prolonged lithium therapy can have adverse effects on renal function. Lithium use has been linked to increased risk of renal impairment (Close et al. 2014; Shine et al. 2015), renal failure (Close et al. 2014) and end-stage renal disease (ESRD) requiring renal replacement therapy (RRT), compared to individuals not using lithium (Close et al. 2014; Aiff et al. 2014). According to recent studies, patients who have been treated with lithium for at least 10 years have an increased risk for CKD (Højlund et al. 2022) and a steeper renal function decline (Fransson et al. 2022).

Other researchers have found no significant impact of stable lithium maintenance therapy on the rate of estimated glomerular filtration rate (eGFR) decline over time (Clos et al. 2015). A comprehensive Danish population-based study has concluded that bipolar disorder is associated with increased risk of CKD independent of the type of treatment (Kessing et al. 2015). Among bipolar patients, both lithium treatment and anticonvulsant treatment were found to be associated with elevated risk of CKD (Kessing et al. 2015). Furthermore, lithium use was not associated with higher risk of ESRD, while anticonvulsant use was (Kessing et al. 2015). These findings were corroborated by a Swedish registry study, which found no increased risk of CKD with lithium treatment compared to valproate treatment (Bosi et al. 2023). In a US study (Pahwa et al. 2021), the age at lithium start, diabetes mellitus, and benzodiazepine use were associated with CKD, while the duration of lithium treatment and hypertension were not. Quantifying the impact of lithium exposure on renal function has proven challenging, partly due to the extended time needed to detect clinically significant outcomes. While epidemiological concepts like incidence, prevalence, and incidence rates are valuable for understanding disease burden and for healthcare resource planning, they are less suitable for explaining individual risks to patients, in order to facilitate informed treatment decisions. In such cases, concepts like cumulative incidence and lifetime risk are more appropriate. Cumulative incidence represents the proportion of individuals developing a specific health outcome within a defined period while lifetime risk quantifies the cumulative risk from a disease-free age to an individual's death. To our knowledge, neither the lifetime risk, nor the age- and time-specific cumulative incidence of severe renal impairment have been previously investigated in lithium-treated patients.

##  Aims

The first objective was to estimate age-specific cumulative incidence and lifetime risk of severe renal impairment, defined as CKD stage 4 or higher (CKD4 +), using data from a large cohort of lithium-treated patients observed for more than 35 years.

Secondly, we hypothesised that the risk of CKD4 + increases with longer lithium exposure and aimed to investigate this association.

## Methods

### Patients and data sources

We identified lithium-treated patients in the Gothenburg area using the laboratory database at the Department of Clinical Chemistry at Sahlgrenska University Hospital in Gothenburg, Sweden. We included individuals who had at least one serum lithium measurement (S-Li) and one serum creatinine concentration (S-creatinine) between January 1, 1980, and December 31, 2009 and met the following criteria:

- age 18 years or older at their first S-Li measurement
- at least one year of laboratory-verified continuous lithium treatment (referred to as *Index treatment*) up to December 31, 2010.
- availability of a *Start creatinine* value, defined as the S-creatinine closest before, on the same day as, or closest after, and within one year from, the start of *Index treatment*.

Individuals with *Start creatinine* levels above the normal reference range were excluded.

We collected patients’ demographics (birth date and sex) as well as laboratory data (S-Li and S-creatinine values and corresponding dates) from January 1, 1980, to December 31, 2017.

Using the Swedish unique identification number, we identified deceased individuals and their date of death up to December 31, 2017, in the Swedish Death Registry (Swedish Death Registry 2018). Individuals undergoing RRT were identified in the Swedish Renal Registry (Swedish Renal Registry 2023).

The psychiatric diagnoses and somatic comorbidities of patients with *Incident CKD4* + were assessed through a structured review of individual health records by two of the authors. The following disorders, requiring chronic medication or hospitalisation, were recorded: cardiovascular conditions, diabetes mellitus, renal and urological diseases, malignancies and others. Cardiovascular disorders encompassed hypertension, angina pectoris, myocardial infarction, arrhythmias, cardiac failure, cerebrovascular diseases, and peripheral vascular diseases. Renal and urological disorders comprised chronic glomerulonephritis, vasculitis, polycystic kidney disease, pyelonephritis with septicaemia, acute renal failure with partial or total recovery, as well as conditions affecting urinary flow and congenital kidney and urinary tract malformations.

### Measurements and data validation

S-Li concentration was determined using flame photometry. S-creatinine was measured using a picrate method until June 1, 2004, and a more specific enzymatic method thereafter. To ensure direct comparability with later measurements, values obtained before June 2004 were adjusted as previously described (Aiff et al. 2015).

As part of data validation, negative S-Li values were excluded. *Start creatinine* values below the lower limit of the reference range were manually analysed by two of the authors. If warranted, these values were disregarded in favour of the next consecutive measurement. Mortality data was cross-checked with laboratory data for consistency, and patients with inconsistent data were excluded.

#### e-GFR calculation

For each S-creatinine measurement, we calculated eGFR using the revised Lund–Malmö formula (Björk et al. 2011; Nyman et al. 2014). This formula incorporates S-creatinine, sex, and age as input values and has been validated as the most accurate eGFR estimator for the Swedish population (Björk et al. 2011).

### Parameter operationalisation

For each individual, we determined the start of *Index treatment*, *Start creatinine*, and *Last creatinine* (the most recent S-creatinine measurement up to and including December 31, 2017).

#### Outcome and disease definition

We defined CKD4 + based on the Kidney Disease Outcomes Quality Initiative (National Kidney Foundation 2002) as eGFR < 30 ml/min/1.73 m^2^ for at least 3 months. CKD4 signifies severe loss of kidney function (approx. 70% or more) and is associated with a high risk of renal failure, heart failure and other complications, like anaemia, hyperkalaemia and mineral disorders. Individuals with two or more eGFR values < 30 ml/min/1.73 m^2^ were manually examined by two of the authors. The CKD4 + diagnosis (*Incident CKD4* +) was assigned to patients with consistent eGFR values below 30 ml/min/1.73 m^2^ for at least 3 months.

#### Exposure definition

We computed effective time on lithium (*Time on Li*) for each individual, defined as the sum of the lithium treatment periods within a specified time interval. Treatment periods were those with at least one S-Li measurement per year, while gaps of one year or more without any S-Li measurements were considered treatment interruptions and not included in the calculation of *Time on Li.* Additionally, we calculated the *Mean S-Li* for each patient, for the time interval considered for the analysis. This was computed as the Area under the S-Li curve (*AUC*) during the treatment periods, divided by the *Time on Li*. An example of these calculations is presented in Additional file 2: Appendix S3.

#### Potential predictor variables

*Start creatinine* values were categorised into three groups (0–2) based on reference intervals (45–90 μmol/l for women and 60–105 μmol/l for men). Categories were defined as follows:

- 0 (lower third of the reference interval, i.e., ≤ 60 μmol/l for women and ≤ 75 μmol/l for men)
- 1 (middle third of the reference interval, i.e., 61–75 μmol/l for women and 76–90 μmol/l for men)
- 2 (upper third of the reference interval, i.e., 76–90 μmol/l for women and 91–105 μmol/l for men).

Categorisation was necessary due to differing reference intervals for S-creatinine between women and men, precluding direct comparisons.

### Study design and analysis

#### Cumulative incidence and lifetime risk of CKD4 + considering the competing risk of death

**Fig. 1:**
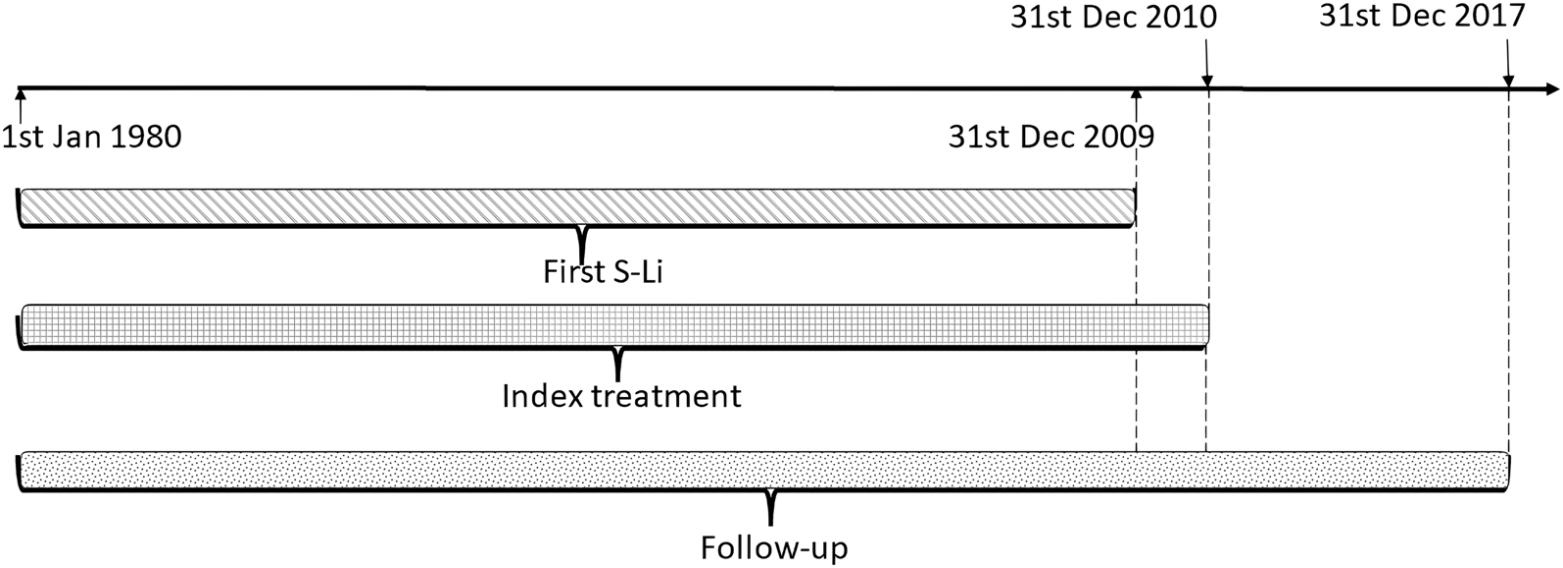
Cohort study timelines

We conducted a retrospective cohort study, comprising all patients included in the primary database. A diagram of the study cohort with relevant timelines is presented in Fig. 1.

To estimate the age-specific cumulative incidence of CKD4 + over 35 years of follow-up while considering competing risks, we employed the Cumulative Incidence Function (CIF) developed by Fine and Gray (Fine and Gray 1999). CIF allows us to calculate the cumulative incidence of an outcome while accounting for competing events.

In absence of competing events, Survival Analysis can be used to estimate the cumulative incidence of a specific outcome, typically expressed as the complement of the Kaplan–Meier curve or as 1 minus the Survival function (Clark et al. 2003). Survival Analysis is primarily designed for analysing time-to-event data within a cohort observed over a defined time period. The individuals experiencing the outcome of interest (such as CKD4 + in our study) are followed until the outcome occurs. Those not experiencing the outcome may either be followed throughout the entire study, or some may be lost to follow-up before the study concludes, and are censored at their last observation (Rich et al. 2010).

In Survival Analysis, censored individuals are assumed to have the same probability of survival as those remained in the study (uninformed censoring). However, if a significant number of censored individuals experience a different (competing) outcome that prevents the occurrence of the outcome of interest, (e.g., dying without developing CKD4 +), this can alter the probability of survival within the censored population.

Failure to account for events that preclude the development of CKD4 + can lead to an overestimation of its cumulative incidence when using the inverse of the Survival function. To address this issue, we used CIF which considers and accounts for the presence of competing risks, yielding more accurate estimates.

Using CIF, the estimate is as accurate as it can get, however, the risk for overestimation is not completely eliminated. Arguably, individuals that are alive but have no more measurements in the database, can be presumed reasonably physically healthy, and having no or low risk for CKD4 + . Those who contribute measurements are either in treatment or had some sort of health problem that needed checked. A lower risk for the outcome among censored individuals compared to those in follow-up may lead to an overestimation of the cumulative incidence.

Individuals with *Incident CKD4* + were followed until the date of this outcome. Individuals without CKD4 + were handled as follows:

- Those alive at the end of the study (December 31, 2017) were censored at the date of their *Last creatinine* measurement.
- The individuals who died during the study were assigned the status of *Competing Death*, if their eGFR values unequivocally indicated that they did not develop *Incident CKD4* + prior to their death. This determination was based on eGFR values present during the last three months of life, which either exceeded 40 ml/min/1.73 m^2^ or were manually reviewed and confirmed not to meet CKD4 + criteria. In cases where eGFR values were absent or uncertain during the last three months of life, the patients were censored at their *Last creatinine* assessment. See Additional file 2: Appendix S4 for details.

To enable age-specific estimates of cumulative incidence and lifetime risk, we categorised age at the start of treatment (*Start age*) into six groups 0–5: 0 (18–34), 1 (35–44), 2 (45–54), 3 (55–64), 4 (65–74), and 5 (≥ 75) years. We sought to achieve a reasonable compromise between intra-group age homogeneity and an adequate number of *Incident CKD4* + in each group. Cumulative incidence over 35-years follow-up for each *Start age* group was presented as both curves and discrete values at 5-year intervals. Lifetime risk for CKD4 + , assuming a lifespan of 90 years, was estimated for *Start age* groups followed up until at least 90 years (i.e., *Start age* groups 55 years and older).

Additionally, we explored the impact of baseline covariates *Sex*, *Start age* and *Start creatinine* (categorised as described), using the Fine-Gray subdistribution proportional hazard model. This model accounts for the competing risk of death and is considered suitable for predicting individual risk (Lau et al. 2009).

#### Association between lithium exposure and incident CKD4 + 

In this phase of the investigation, we designed a matched 1:4 case–control study, with Cases comprising individuals who developed *Incident CKD4* + . Matching was based on the calendar year of *Incident CKD4* + (referred to as *Matching year*).

To facilitate the matching process, we categorised Cases' age at the *Matching year* into four *Matched age* groups 0–3: 0 (40–59), 1 (60–69), 2 (70–79), and 3 (≥ 80) years. We aimed to ensure age homogeneity within each category, while minimising the number of categories. No *Incident CKD4* + cases were observed below 40 years, which is why we did not include *Matched Age* categories younger than 40. Note that *Matched age* is the age at *Incident CKD4* + , and should not be confused with *Start age* as described and used in the previous analysis, which represents the age at start of *Index treatment*.

For each Case, we compiled a pool of potential Controls who shared the same sex and belonged to the same *Matched age* group as the Cases. Furthermore, the Controls had at least one S-creatinine measurement during the *Matching year* and had not experienced *Incident CKD4* + up to and including that year. Subsequently, from each pool, we randomly selected four Controls for each Case, employing a computerised randomization method. This selection process allowed for the possibility of the same individual serving as a control for multiple cases and for cases to be selected as controls before developing CKD4 + .

**Fig. 2:**
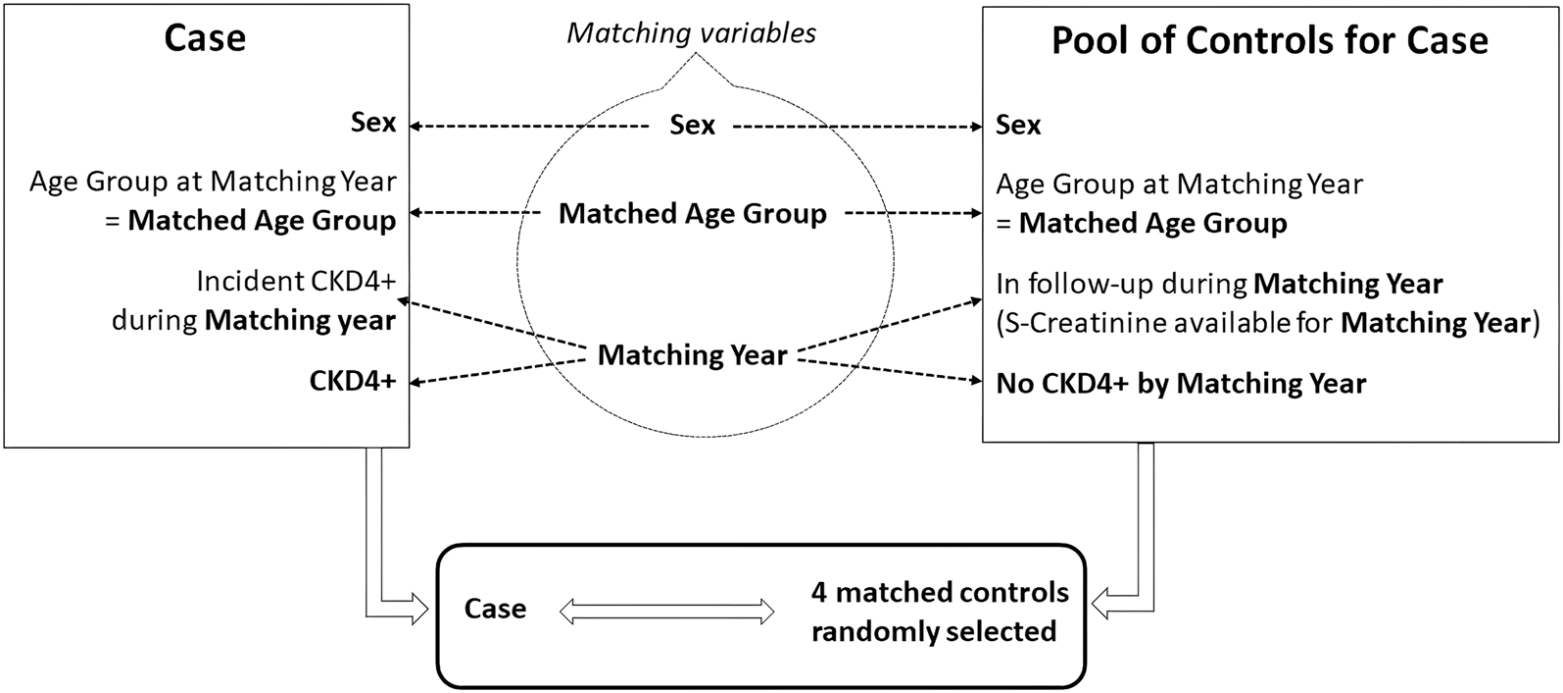
Selection process for the Case Control study

A visual representation of the selection process is provided in Fig. 2.

*Time on Li* and *Mean S-Li* were computed for each participant, from the start of *Index treatment* up to and including the *Matching year*.

For comparing means, independent samples t-test was used for variables with normal distribution and Mann–Whitney U test for variables with non-normal distribution. For categorical variables, Pearsson’s chi-squared test was utilised.

To investigate the relationship between *Incident CKD4* + and lithium exposure, we employed logistic regression. *Time on Li*, as the measure of lithium exposure, was tested both as a continuous variable and categorised according to various patterns. We chose the categorization into three groups 0–2: 0 (≥ 1 and < 5), 1 (≥ 5 and < 20), and 2 (≥ 20) years, which minimises the number of categories while ensuring intra-category consistency in terms of the strength of association. In addition to the matching variables (*Sex*, *Matching year* and categorised *Matched age*), we adjusted for *Start creatinine* (categorised as previously described). The inclusion of matching variables enabled the use of standard logistic regression available in the SPSS program, instead of conditional logistic regression, which is typically recommended for matched case–control studies (Pearce 2016; Wan et al. 2021).

A two-sided p-value less than 0.05 was considered statistically significant.

### Software

Microsoft Excel 2010 (Microsoft Corp, Redmond, Washington, USA) and MatLab 2019a (MathWorks, Natick, Massachusetts, USA) were used for data processing and for randomization. SPSS Statistics v. 28.0.1.0 (IBM Corp, Armonk, NY, USA) was used for descriptive statistics, comparison of means and proportions, as well as for logistic regression. R version 4.3.1 in conjunction with the package cmprsk version 2.2-11 was used for Cumulative Incidence estimates and variance calculations, as well as for implementing the Fine Gray subdistribution hazards model. In addition, Python 3.7.9, along with the packages numpy version 1.25.0, pandas version 2.0.1, and matplotlib 3.7.0 was used to plot the Cumulative Incidence curves.

During the preparation of this work the authors have used ChatGPT-3.5 in order to enhance the text’s grammar, style and clarity. After employing this tool, the authors reviewed and edited the content as needed and take full responsibility for the content of the publication.

## Results

### Cumulative incidence and lifetime risk of CKD4 + considering the competing risk of death

**Fig. 3:**
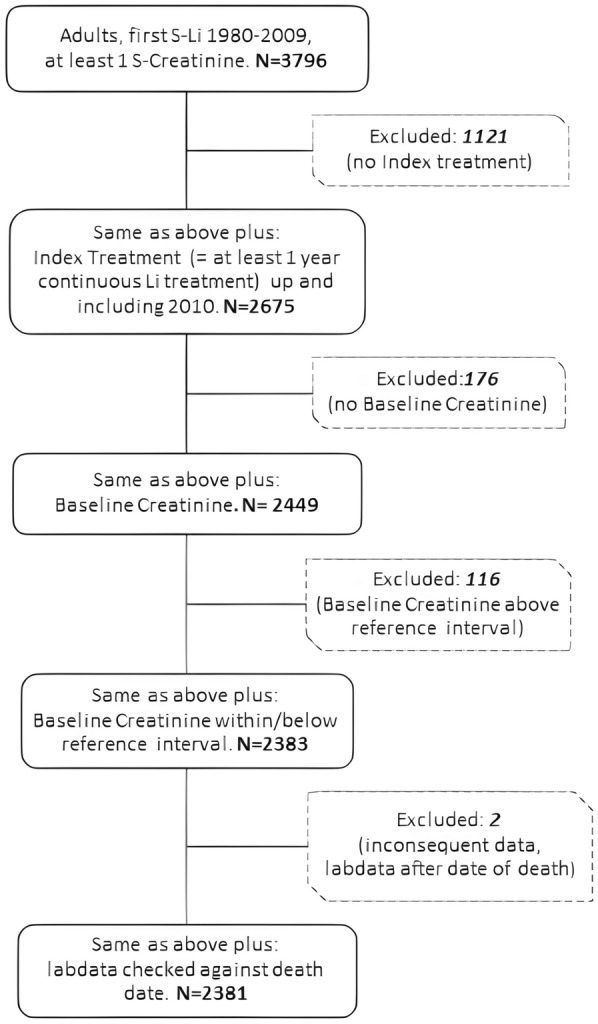
Study flow-chart

The study database included 2381 patients, all of which were included in the cohort study. A flow-chart illustrating the selection process is presented in Fig. 3.

The descriptive statistics of the cohort are presented in Table 1.

**Table 1:** Descriptive statistics of the cohort

Categorical variables	Category	Frequency	Percent
Sex			
	0 (Men)	931	39.1
	1 (Women)	1450	60.9
Start age			
	0 (18–34 years)	625	26.2
	1 (35–44 years)	521	21.9
	2 (45–54 years)	488	20.5
	3 (55–64 years)	382	16.0
	4 (65–74 years)	256	10.8
	5 (≥ 75 years)	109	4.6
Start creatinine			
	0 (lower third)	1128	47.4
	1 (middle third)	974	40.9
	2 (upper third)	279	11.7
CKD4 +		103	4.3
RRT		15	0.6
Competing deaths		499	21.0
Deaths treated as censorings		385	16,2

The Mean *Start age* was 47.1 years with Standard Deviation (SD) 15.7 years, Mean observation time was 14.9 (SD 8.4) years, Mean *Time on Li* was 9.7 (SD 7.2) years and *Mean S-Li* 0.60 (SD 0.10) mmol/l. The study totalled 35453 patient observation years, of which 23120 years of lithium treatment.

The main psychiatric diagnosis and somatic comorbidities of individuals with *Incident CKD4* + are presented in Table 2.

**Table 2:** Psychiatric diagnoses and somatic comorbidities for individuals with *Incident CKD4* +

Psychiatric diagnosis	Frequency	Percent of all cases	Percent of known
Bipolar disorder	59	57.3	83.1
Major depression disorder (recurrent)	7	6.8	9.9
Schizoaffective disorder	4	3.9	5.6
Psychosis	1	1.0	1.4
Missing data	32	31.0	NA
Total	103	100	NA
**Disease Category**	**Frequency**	**Percent of all cases**	**Percent of known**
Cardiovascular Disorders (hypertension included)	50	48.5	50
Diabetes mellitus	20	19.4	20
Primary renal and urological conditions	18	17.5	18
Malignancies	25	24.3	25
Any somatic comorbidity	84	81.6	84
Missing data	3	2.9	NA

**Fig. 4:**
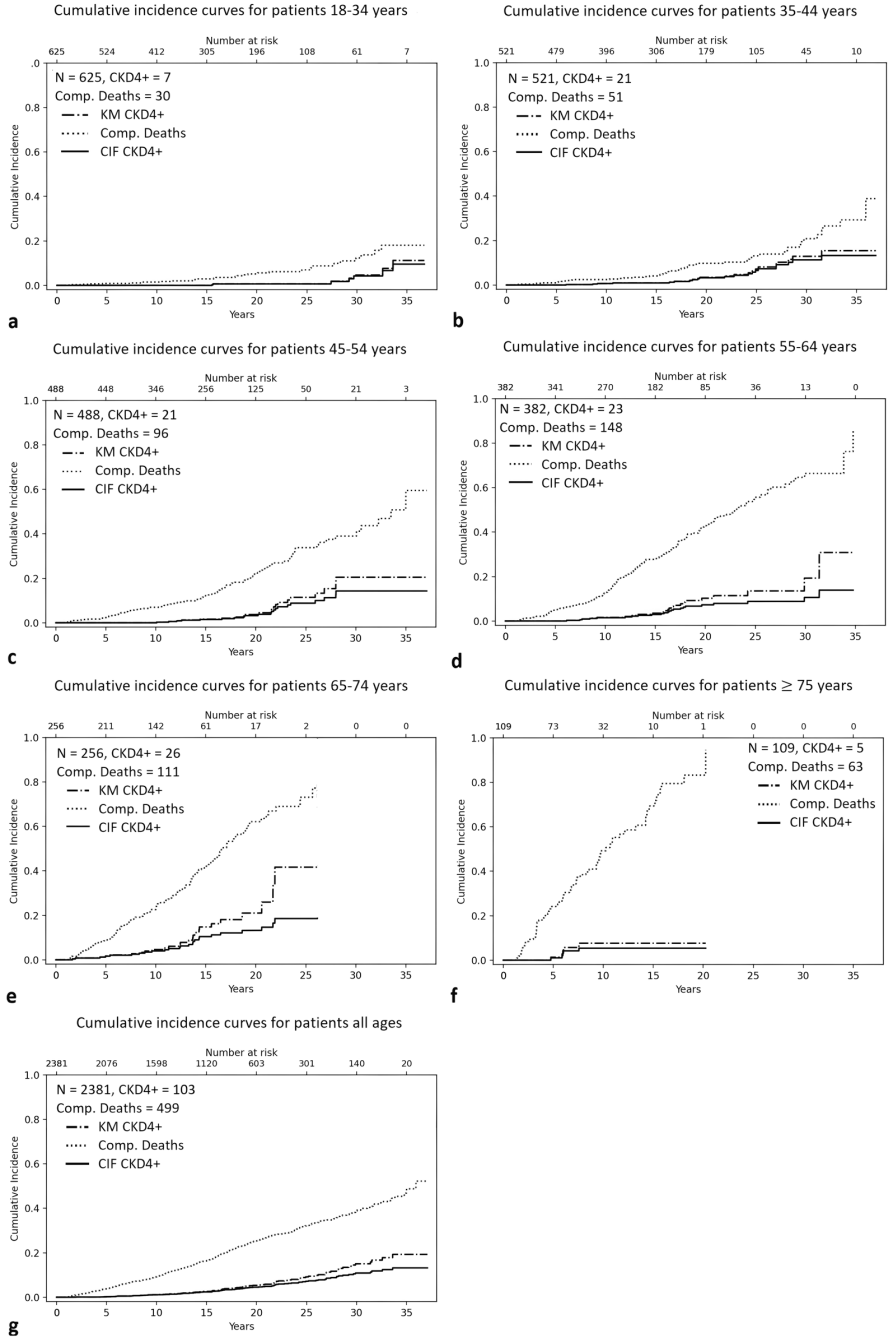
Cumulative Incidence curves by *Start age* group. Legend: *N* total number of individuals in the group; *CKD4 +*  number of *Incident CKD4* + in the group; *Comp Deaths* Number of competing deaths / Cumulative incidence of competing deaths; *KM CKD4* +  inverse of Kaplan Meier curve for *Incident CKD4* + ; *CIF CKD4* +  Cumulative Incidence Function for *Incident CKD4* + , considering the risk of competing deaths

The cumulative incidence curves of the study population by *Start age* group are presented in Fig. 4a–f. Figure 4g presents the cumulative incidence in the whole patient material. Of the three curves presented on each diagram, CIF CKD4 + (the solid line) illustrates the cumulative incidence function for CKD4 + , considering competing deaths.

The cumulative incidence of CKD4 + , taking into consideration the competing risk of death, at 5-year intervals, stratified by *Start age* group, is presented in Table 3.

**Table 3:** Cumulative Incidence of CKD4 + by *Start age* group

	Estimate % (SD%)						
Start age	Follow-up time (years)						
	5	10	15	20	25	30	35
0 (18–34 years)	0.0 (0.0)	0.0 (0.0)	0.0 (0.0)	0.6 (0.5)	0.6 (0.5)	4.2 (2.1)	9.6 (4.2)
1 (35–44 years)	0.0 (0.0)	0.7 (0.4)	0.9 (0.5)	3.2 (1.0)	6.5 (1.8)	11.3 (2.7)	13.3 (3.3)
2 (45–54 years)	0.0 (0.0)	0.0 (0.0)	1.5 (0.7)	3.2 (1.1)	8.8 (2.2)	14.3 (3.4)	14.3 (3.4)
3 (55–64 years)	0.0 (0.0)	1.5 (0.7)	2.9 (1.0)	7.3 (1.6)	8.8 (2.0)	10.6 (2.6)	13.9 (4.1)*
4 (65–74 years)	1.6 (0.8)	4.0 (1.3)	10.4 (2.3)	13.2 (2.8)	18.6 (4.0)*		
5 (≥ 75 years)	1.0 (1.0)	5.4 (2.4)	5.4 (2.4)*				
All ages	0.2 (0.1)	1.1 (0.2)	2.4 (0.4)	4.5 (0.6)	7.2 (0.8)	10.9 (1.3)	13.2 (1.7)

SD = Standard Deviation of the estimate; * Cumulative incidence equals lifetime risk for a lifetime horizon of 90 years

*Start age* groups 55–64, 65–74, and ≥ 75 years were followed up 35, 25, and respectively 15 years, time by which the youngest patients in the respective group will have reached 90 years. Thus, their lifetime risk, considering a lifetime horizon of 90 years, equals their cumulative incidence at the end of follow-up. For younger patients, lifetime risk could not be estimated, as they were not followed up long enough.

We further analysed the effects of baseline covariates, including *Sex*, *Start age* group, and *Start creatinine* (categorised as previously described), using the Fine-Gray subdistribution hazards model. The results are presented in Table 4.

**Table 4:** Results of the Subdistribution Hazards model

				95%CI for HR		
Variable	Category	Reference	HR	Lower	Upper	p-value
Sex	0 (men)	REF				
	1 (women)		1.18	0.77	1.81	0.440
Start age	0 (18–34 years)	REF				
	1 (35–44 years)		3.20	1.37	7.45	0.007
	2 (45–54 years)		3.18	1.37	7.39	0.007
	3 (55–64 years)		3.80	1.64	8.79	0.002
	4 (65–74 years)		8.25	3.60	18.90	< 0.001
	5 (≥ 75 years)		3.29	1.02	10.64	0.047
Start creatinine	0 (lower third)	REF				
	1 (middle third)		2.02	1.29	3.17	0.002
	2 (upper third)		4.60	2.75	7.68	< 0.001

HR = Hazard Ratio. 95%CI = 95% Confidence Interval. REF = the reference category in each categorical variable. Number of patients included in the model: 2381. Outcome variable: *Incident CKD4* + . Predictor variables: *Sex*, *Start age* (categorised) and *Start creatinine* (categorised)

Among the analysed covariates, *Start age* and *Start creatinine* demonstrated predictive value, while *Sex* did not contribute significantly to the model’s prediction.

### Association between lithium exposure and incident CKD4 + 

From the initial cohort of 2381 patients included in the first analysis, a subset of 103 patients met the criteria for *Incident CKD4* + . Each of these individuals was matched with four controls, as outlined in the section "Study design and analysis".

A total of 515 patients, comprising 103 Cases and 412 Controls, were included in the matched 1:4 Case–Control study. The descriptive statistics for both groups (Cases and Controls) are detailed in Table 5.

**Table 5:** Descriptive statistics of 103 Cases and 412 Controls

		Cases (N = 103)		Controls (N = 412)		p
Continuous variables		Mean	Std. Deviation	Mean	Std. Deviation	
Start age (years)		55.51	13.74	57.84	12.81	0.105^a^
S-Li (mmol/l)		0.61	0.09	0.59	0.10	0.138^a^
**Categorical variables**	**Category**	**Frequency**	**Percent**	**Frequency**	**Percent**	**p**
Start creatinine						< 0.001^b^
	0 (lower third)	31^d^	30,1	213^c^	51,7	
	1 (middle third)	43^c^	41,7	158^c^	38,3	
	2 (upper third)	29^d^	28,2	41^c^	10,0	
CKD4 + by Matched age group						
	0 (40–59 years)	9	8.7			
	1 (60–69 years)	29	28.2			
	2 (70–79 years)	40	38.8			
	3 (≥ 80 years)	25	24.3			

^a^Normally distributed variables, independent samples t-test was used for comparison of means

^b^Categorical variable, Pearsson’s chi-squared test was used for comparison of proportions

^c,d^Each letter denotes a subset of categories of which column proportions do not differ significantly from each other at the 0.05 level

Matching variables *Sex* and *Matched age* group were identical in Cases and Controls (descriptive statistics for matching variables are presented in Additional file 2: Appendix S5). There was no significant difference between Cases and Controls with regard to *Start age* and *Mean S-Li*. However, *Start creatinine* differed significantly between Cases and Controls: more patients among Cases had a *Start creatinine* within the upper third of the reference range, and less within the lower.

The results of the logistic regression employed to investigate the association between *Time on Li* and *Incident CKD4* + , are presented in Table 6. We observed a strong correlation between *Time on Li* and the total lithium exposure, as measured by *AUC* (refer to Additional file 2: Appendix S6 for details). We selected *Time on Li* as a measure for lithium exposure due to its simplicity and easy accessibility for both physicians and patients. The regression equation was adjusted for the matching variables and for the predictor variable *Start creatinine* (matching variables were not associated with the outcome and are not presented in Table 6; the full model, including matching variables is presented in Additional file 2: Appendix S7).

**Table 6:** Results of logistic regression

Variable	Category	Reference	OR	95% CI for OR		p-value
				Lower	Upper	
Time on Li						< 0.001
	0 (1 ≤ x < 5 years)	REF				
	1 (5 ≤ x < 20 years)		2.29	1.25	4.19	0.007
	2 (x ≥ 20 years)		5.85	2.54	13.44	< 0.001
Start creatinine						< 0.001
	0 (lower third)	REF				
	1 (middle third)		2.15	1.27	3.64	0.004
	2 (upper third)		6.84	3.55	13.18	< 0.001

*OR* Odds Ratio. *95%CI* 95% Confidence Interval. *REF* the reference category in each categorical variable. Number of patients included in the model: 515. Outcome variable: *Incident CKD4* + . Predictor variable: *Time on Li* (categorised). Adjusted for *Start Creatinine* (categorised) and matching variables *Sex*, *Matching year* and *Matched age* group. Matching variables not presented. Predictive efficiency: 79.8% (cut-off value 0.5). Hosmer–Lemeshow test p = 0.799 (no evidence that the model is a poor fit for the data)

The results indicate a statistically significant association between *Time on Li* and *Incident CKD4* + . In the partly adjusted model (adjusted for matching parameters only), the strength of the association decreased, but the significance was maintained for both *Time on Li* 5–20 years (OR 1.8, 98% CI 1.003–3.13, p = 0.049) and Time on Li ≥ 20 years (OR 3.52, 95% CI 1.62–7.65, p = 0.001. In the unadjusted model, only *Time on Li* ≥ 20 years was significantly associated with increased risk for CKD4 + (OR = 2.99. 95% CI: 1.46–6.12. p = 0.003). For Time on Li 5–20 years, the association was not statistically significant (OR = 1.66, 95% CI: 0.95–2.90, p = 0.073).

The association between *Time on Li* and *Incident CKD4* + , with and without adjustments, remained consistent across all alternative categorization patterns tested for *Time on Li* (including three, four and five *Time on Li*-categories), and when considering *Time on Li* as a continuous variable. These results indicate a robust association.

The results of adjusted and unadjusted models including alternative categorisation patterns are presented in Additional file 2: Appendix S8.

## Discussion

The main reason to undertake this study was the perceived need for better quantifying the presumptive long-term renal risks associated with lithium treatment, in terms that would make sense for a patient. In Sweden, healthcare legislation requires healthcare professionals to give individually adapted information about (among others) risks and benefits of available treatments, enabling shared decision-making. Lithium is perceived by a number of physicians and researchers as being underused (Zivanovic 2017), and this is a result of several factors: clinicians’ reluctance to initiate lithium treatment, treatment termination due to side effects (Öhlund et al. 2018), but also patients’ refusal to accept lithium. A recent survey exploring clinicians' attitudes toward lithium use in bipolar disorders (Hidalgo-Mazzei et al. 2023) reported that over 70% of respondents (886 clinicians in 43 countries) considered lithium as their first choice for maintenance medication in bipolar disorder. However, 55% of them expressed concerns about renal function alterations. The primary reasons for clinicians' reluctance to prescribe lithium were patients' negative beliefs or attitudes toward lithium, followed by concerns about long-term side effects and safety.

A noticeable discrepancy exists between the statements of surveyed physicians and real-world practices. Data from a large multinational bipolar cohort of 10,351 patients across North America, Europe and Australia revealed that lithium was prescribed to only 29% of the patients (Singh et al. 2023). These findings reinforce the concerns about the widespread under-prescription of lithium (Zivanovic 2017; Malhi et al. 2023). Unclear or difficult to comprehend information about potential risks may lead to an exaggerated negative perception of lithium, both among clinicians and patients. Meaningful patient information about the long-term renal effects of lithium should ideally include (among others): what is the risk for severe renal impairment for the actual patient and what is the time perspective considered? What are the possible consequences? What are the benefits of lithium? What are the alternatives?

In this respect, one of the study aims was to estimate cumulative incidence and lifetime risk for CKD4 + in lithium-treated patients in a way that would allow an easy-to-understand comparison with the general population. The lifetime risk is expressed in relation to a defined outcome-free start age and requires a follow-up time long enough to cover the age that may reasonably approximate life expectancy. In Sweden, the current life expectancy is nearly 85 years for women and just over 81 for men (Statistics Sweden 2022), hence 90 years of age was considered an appropriate lifetime horizon for estimation of lifetime risk. In that matter, the lifetime risk of CKD4 + was: 13.9%, 18.6%, and respectively 5.4% for *Start age* groups 55–64, 65–74, and ≥ 75 years.

These figures cannot be evaluated in isolation, what we want to know is the magnitude of the excess risk attributable to lithium. However, to our knowledge, data on lifetime risk for CKD in the general population is quite sparse and in bipolar patients not treated with lithium—not available at all. In a simulation study from the USA (Grams et al. 2013), the residual lifetime risk for CKD4 + for a lifetime horizon of 90 years was estimated at: 10.2–10.5% for white men of age 60–80 years and respectively 11.1–11.8% for white women of the same age. European data is available only from a prospective longitudinal study from Iceland (Inker et al. 2015). The estimates in the latter study were much lower (3.4–3.7% for men and 3.2–3.6% for women, aged 45–75 years). However, the results are not comparable, as the lifetime horizon considered in the Icelandic study was shorter (85 years) and the follow-up controls, upon which the CKD4 diagnosis was based, were scheduled at 3–7 years intervals, thus probably missing an important number of CKD4-cases. Interestingly, in the only group followed up until 90 years (patients aged 80), the lifetime risk was 7% for both women and men, close to the results in the American simulation study.

While direct comparisons with American figures (Grams et al. 2013) have limitations, this study serves as the sole available reference in the general population. Notably, within our study population, the lifetime risk of CKD4 + among those aged 55–75 exceeded that observed in the American study (Grams et al. 2013). This outcome was in line with expectations and aligns with prior research (Close et al. 2014; Shine et al. 2015; Van Alphen et al. 2021).

When assessing this excess risk, a few factors come into play. First, as detailed in the Methods section, some potential for overestimation persists. Second, it is important to acknowledge that a portion of the excess risk may be associated with other risk factors. Specifically, our CKD4 + group exhibited a significant burden of somatic diseases, as indicated in Table 2. Of the 103 patients who developed CKD4 + , only 19 (18.4%) had no recorded somatic comorbidities. This data implies that the increased incidence of CKD4 + should not be solely attributed to lithium; somatic comorbidities likely made a substantial contribution (Forty et al. 2014).

However, the lifetime risk for individuals starting lithium at ≥ 75 years was much lower. As renal impairment usually progresses slowly, often over many years, a possible explanation might be that these older individuals simply did not live enough to progress to CKD4 + , a large proportion of them having died of other causes, precluding *Incident CKD4* + . As shown in Table 3, their cumulative incidence was 5.4% already at 10 years follow-up (highest among all *Start age* groups), but it didn’t increase thereafter. On the other hand, the *Start age* group ≥ 75 years is the smallest group, with the lowest number of individuals and outcomes, and the lowest precision of the estimates (large SD’s, as indicated in Table 3), thus estimate error may, in part, account for the low figure.

The available data did not allow to estimate the lifetime risk of CKD4 + in patients starting lithium earlier than 55 years (as their age at end of follow up was lower than 90 years). However, it showed that their risk for CKD4 + is essentially zero the first ten years after commencing lithium (see Fig. 4a–f and Table 3): at five years follow-up, their cumulative incidence for CKD4 + was zero, and at ten years it marginally increased (to 0.7%) only for the *Start age* group 35–44 years, and remained zero for the other two groups.

Our study does not provide specific guidance on managing patients with an accelerated decline in renal function, a matter of paramount clinical importance. The central question at hand is whether to continue or discontinue lithium treatment. If we acknowledge that lithium poses a risk for CKD, continuing its use could potentially worsen renal decline. On the other hand, discontinuing lithium may raise the risk of relapse (Kumar et al. 2023), which may have severe consequences, including the risk of suicide (Gitlin 2023), and offers no guarantees of renal function improvement. It is worth noting that other psychopharmaceuticals may also have adverse effects on the kidneys (Kessing et al. 2015; Bosi et al. 2023; Højlund et al. 2020).

While some evidence suggests that continuing lithium treatment may not necessarily lead to further deterioration of renal function toward ESRD (Pahwa et al. 2021; Kumar et al. 2023; Kessing et al. 2017; Pahwa and Singh 2022), cases of progression to ESRD have been reported (Gitlin 2023). Also, some evidence exists, albeit limited, that discontinuing lithium may improve renal function at least in some patients (Hoekstra et al. 2022). 

In summary, there is no definitive answer, no “one-size-fits-all”-recommendation, and managing patients with progressive renal function decline is arguably one of the most challenging aspects of the lithium treatment.

In our previous research (Golic et al. 2023), we found that the pre-treatment creatinine value, albeit within the reference interval, was a prognostic indicator for *Incident CKD4* + over 10-years follow-up. The current results show that *Start creatinine* level remained prognostic even when the follow-up period was extended to more than 3 decades. Specifically, *Start creatinine* in the upper third of the reference range was strongly associated with a higher risk of CKD4 + compared to the lower third.

In the matched case–control study, as all our study participants were lithium-treated, we categorised the *Time on Li* and used the lowest exposure (*Time on Li* 1–5 years) as reference. We found an association between the length of lithium exposure and *Incident CKD4* + , with larger OR for increased exposure (see Table 6). However, it is important to acknowledge that the use of OR as a measure of risk has its drawbacks. The only use of OR in medicine is as an approximation of risk, but the approximation is good only for rare diseases. What is a “rare disease” in this context is yet another question to which there is no clear answer. Opinions differ, some authors (Davies et al. 1998), consider that the disease rate should fall below 20%, others (Chen et al. 2010)—below 10%, for a reasonable “safe” use of OR. Regardless of the disease rate, the OR will always overestimate the risk ratio (RR) to some degree, but serious divergences between OR and RR occur only with large effects on groups at initial high risk (Davies et al. 1998). In order to avoid drawing exaggerated conclusions from studies using logistic regression, a method for interpreting OR was proposed (Chen et al. 2010), where OR below 1.5 indicates a weak association (small size effect) and higher than 5—strong association (large size effect). Hence, our findings suggest that lithium exposure of 20 years or more is associated with a substantial increase in the risk of CKD4 + , while the association was only moderate for lithium exposure of 5–20 years.

How do the present results compare to previous research? The association between the duration of lithium treatment and impairment of kidney function has been previously explored using “softer” outcomes. A Swedish study (Aiff et al. 2015) found a continuous increase in S-creatinine with the lithium treatment duration. A Dutch study (Van Alphen et al. 2021) found that the duration of lithium exposure was associated with the risk for CKD3 (although the OR suggests a small size effect). A more recent Swedish study (Fransson et al. 2022) found that bipolar and schizoaffective patients who have used lithium for more than 10 years had a steeper eGFR decline compared to both bipolar/schizoaffective patients with 0–10 years of lithium exposure and to a reference population. A recent Danish register study (Højlund et al. 2022) found that lithium was associated with increased risk for CKD, with stronger association for more than 10 years of use. These results, although not directly comparable with the present study, point in the same direction, namely that increased exposure to lithium is associated with decreased glomerular function and increased risk for CKD. However, not all research findings are consistent. A US study, involving 154 bipolar patients treated with lithium and followed for up to 9 years, found no association between the duration of lithium exposure and incident CKD stage 3 or higher (Pahwa et al. 2021).

It is worth noting that in all *Start age* groups, the risk of death from other causes was numerically larger than the risk for CKD4 + (see Fig. 4). Even in the youngest *Start age* group (18–35 years), the number of competing deaths outnumbered *Incident CKD4* + by more than 4:1.

In this group of individuals receiving lithium, the risk of developing CKD4 + was primarily associated with older age. The age at which CKD4 + was diagnosed is as follows: 25 patients (24.3%) were 80 years or older, 40 patients (38.8%) were aged 70–79, 29 patients (28.2%) were aged 60–69, and 9 patients (8.7%) were aged 40–60. Notably, there were no cases among individuals under 40 years of age. The incidence pattern of CKD4 + observed in this lithium-treated population closely resembles that reported in a population-based study in the Netherlands (Blijderveen et al. 2014).

Over the past couple of decades, there has been a growing debate about the definition of CKD when age-independent criteria are applied. Critics argue that these criteria may erroneously label the natural, age-related decline in renal function, which typically begins around the age of 50, as a medical condition. Consequently, this could lead to the overdiagnosis of many asymptomatic elderly individuals, who might remain in CKD stage 3 throughout their lives.

Some experts (Delanaye et al. 2019) have proposed an age-adapted CKD definition, suggesting a pathological threshold of 45 ml/min/1.73 m^2^ for individuals aged 65 and older, and 75 ml/min/1.73 m^2^ for those under 65. This age-specific approach is seen as more medically and socially sensible. It advocates for greater attention to be directed toward younger patients with 'milder' CKD since they are at risk of disease progression and early mortality (Kula et al. 2023). By focusing on this younger demographic, based on the recommended thresholds, it becomes possible to proactively manage associated risk factors. This approach holds promise for potential developments in lithium monitoring guidelines as well.

### Strengths and limitations

To our knowledge, this is the first study to include a large number of unselected lithium-treated patients followed up to more than 35 years. The diagnosis of CKD4 + based on laboratory data rather than health charts ensured a high level of accuracy, as chart-diagnosis might be delayed or even missing in asymptomatic patients. The outcomes are expressed in easy-to-grasp concepts and the findings may have direct clinical applicability for patient communication and clinical decision making.

The study has a number of limitations. The risk of surveillance bias has been previously discussed (Kessing et al. 2015; Nielsen et al. 2018; Wiuff et al. 2023), whereby regular renal function monitoring in lithium patients is likely to result in a higher detection of asymptomatic CKD, compared to patients that are not monitored. This risk might be less important though when the outcome is as severe as CKD4 + .

The risk of overestimation of cumulative incidence and lifetime risk, due to possible uninformed censoring, has been mentioned before and must not be overlooked.

A number of assumptions were made in relation to parameter operationalization such as: one year without any S-Li was regarded as treatment discontinuation, and the presence of S-Li at least once a year was regarded as a treatment period. This may not necessarily reflect the reality, as some patients may have taken lithium without proper treatment monitoring. Patients' follow-up started with the first year of continuous treatment, according to our operational definitions. In reality, some patients may have had a longer lithium treatment than the one computed in our analysis, and this may have introduced a bias.

An important limitation of this study is the lack of complete data on somatic comorbidities and concurrent medications. The regression models were not adjusted for these factors, which are known to be associated with CKD in the general population (Low et al. 2015; Zeng et al. 2023) and in lithium-treated patients (Shine et al. 2015; Rej et al. 2014; Aiff et al. 2019). While it is unlikely that adjusting for somatic comorbidities would invalidate the results, such an adjustment would underscore the potential contribution of these risk factors more clearly.

The number of *Incident CKD4* + was 103 in the whole material of 2381 patients. This allowed for multivariate analyses with categorised variables, observing the statistical rule of thumb of 10 outcomes per parameter. However, the *Start age* groups were comparatively small, and the number of *Incident CKD4* + per group was low (in particular the youngest and the oldest *Start age* group), resulting in relatively low precision for the Cumulative Incidence estimates, as indicated by the comparatively large standard deviations presented in Table 3. Statistically meaningful sub-analyses (i.e., by sex) were not possible due to the low number of *Incident CKD4* + among men (only 31).

The correlation between *Time on Li* and *Incident CKD4* + was examined only within the lithium-treated population. Comparison was made between individuals with larger exposures and those with a reference exposure (*Time on Li* 1–5 years), but not with lithium-free individuals. We, therefore, did not explore whether the risk for CKD4 + is higher for our reference exposure (1–5 years) compared to lithium non-users.

The patients recruited in our study have been followed up and managed by Swedish physicians, acting in accordance with national and local monitoring guidelines. S-Li has been well kept within therapeutic limits, and the lithium treatment has been terminated in a number of patients. The local guidelines and routines, clinicians’ prescribing practice, patients’ medical literacy, compliance, and degree of involvement in the decision-making process are likely to influence the characteristics of the study population, the length of the treatment, the target serum lithium level and / or the outcomes. Hence, the results may only be generalizable to settings with similar lithium monitoring guidelines and recommendations, access to laboratory testing and socio-demographics.

## Conclusions

Of 2381 lithium-treated patients, with a Mean *Start age* of 47,1 years and a cumulative follow-up of 35453 patient-years, 103 (4.3%) met the criteria for CKD4 + and only 15 (0.6%) reached ESRD and subsequently received RRT.

Lifetime risk for CKD4 + for patients starting lithium at 55–74 years old was 13.9–18.6% for a lifetime horizon of 90 years. A risk of overestimation is inherent to the estimation method, however, our lifetime estimates were higher compared to US data on general population (Grams et al. 2013) for the same lifetime horizon. Not all excess risk can be attributed to lithium; part of it may be linked to a high burden of somatic comorbidities and lifestyle factors.

The *Start age* group 65–74 years appears to be most vulnerable (lifetime risk 18.6%), while patients initiating lithium treatment at 75 years and older had a lower lifetime risk of CKD4 + , of only 5.4%. Estimate error and higher risk of death of other causes might partly explain the low figure for the oldest patients.

The incidence of CKD4 + for individuals starting lithium 18–54 years was zero during the first five years and remained very low for the first ten years after treatment start.

The majority of *Incident CKD4* + cases occurred in elderly individuals, with only 8% occurring at age 40–60 years, and 63% after the age of 70.

Duration of lithium treatment of 20 years or more was strongly associated with increased risk of CKD4 + , compared to lithium exposure of 1–5 years. *Time on Li* between 5 and 20 years was only moderately associated with increased risk for CKD4 + . *Start creatinine* in the upper range of the reference was a risk predictor.

The marked differences observed in the age-related pattern of incidence highlight the importance of specifying age and time perspective, if research results are to be helpful in making individual risk predictions. The aggregated estimates on the whole patient material are not useful for this purpose.

Consistent with previous research, our results indicate that lithium presents a risk factor for CKD4 + . This should be a matter of consideration, however, it does not motivate refraining from initiating lithium in patients who might benefit from it. A therapeutic trial makes possible the evaluation of lithium’s benefits, without posing significant renal risks for a large majority of patients, in particular the ones below 55 years.

Regular monitoring of renal function and lithium levels remain crucial for early detection of log-term side effects. As pointed out by other authors (Fransson et al. 2022), assertive management of other known renal risk factors (such as diabetes and hypertension) is mandatory.

While this study offers some insights that might be useful for informed decision-making in clinical practice, it also highlights the need for further research, in order to refine risk assessment for different patient groups and find strategies that minimise the risks while optimising the potential therapeutical benefits.

### Supplementary Information

**Additional file 1: Appendix S1. **STROBE Statement—checklist of items that should be included in reports of observational studies

**Additional file 2: Appendix S2.** List of abbreviations. **Appendix S3.** Example of computing Time on Li and Mean S-Li, using Area under the curve (AUC). **Appendix S4.** Identification of competing deaths. **Appendix S5.** Descriptive statistics of the matching variables for the case-control study. **Appendix S6.** Rationale for choosing Time on Li as measure for lithium exposure. **Appendix S7.** Results of logistic regression in the chosen model, including the matching variables. **Appendix S8.** Results of logistic regression for alternative categorisations of Time on Li.

## Data Availability

The data supporting this study cannot be made publicly available due to lack of ethics committee permission, as data-sharing was not part of the approval process. The coding specifications are available from the corresponding author upon reasonable requests.

## Abbreviations

CIF: Cumulative incidence function
CIF CKD4 +: Cumulative incidence function for incident CKD4 + , considering the competing risk of death
CKD: Chronic kidney disease
CKD4: Chronic kidney disease stage 4
CKD4 +: Chronic kidney disease stage 4 or higher
Comp. Deaths: Number of competing deaths/Cumulative Incidence of competing deaths
eGFR: Estimated glomerular filtration rate
ESRD: End stage renal disease
HR: Hazard ratio
KM CKD4 +: Inverse of Kaplan Meier curve for Incident CKD4 +
OR: Odds ratio
RRT: Renal replacement therapy
RR: Risk ratio
S-Creatinine: Serum creatinine concentration
S-Li: Serum lithium concentration
SD: Standard deviation
