# Supplementary material for: Lifetime risk of severe kidney disease in lithium-treated patients: a retrospective study
Source: Int J Bipolar Disord. 2023 Dec 9;11:39. doi: 10.1186/s40345-023-00319-2 (PMC10710395; doi:10.1186/s40345-023-00319-2)
Supplement: Supplementary file 1 — Additional file 1: Appendix S1. STROBE Statement—checklist of items that should be included in reports of observational studies [file 40345_2023_319_MOESM1_ESM.docx]

**Appendix 1** - STROBE Statement—checklist of items that should be included in reports of observational studies

|  | Item No. | Recommendation | | Relevant text from manuscript |
| --- | --- | --- | --- | --- |
| **Title and abstract** | 1 | (*a*) Indicate the study’s design with a commonly used term in the title or the abstract | | See Title. Two studies are presented: a cohort study and a case-control study. The title would become too long if all relevant information would be included. |
|  |  | (*b*) Provide in the abstract an informative and balanced summary of what was done and what was found | | See Abstract |
|  | | |  |  |
| Background/ rationale | 2 | Explain the scientific background and rationale for the investigation being reported | | See Background |
| Objectives | 3 | State specific objectives, including any prespecified hypotheses | | See “Aims”, under “Methods” |
|  | | |  |  |
| Study design | 4 | Present key elements of study design early in the paper | | See “Study design and dnalysis” |
| Setting | 5 | Describe the setting, locations, and relevant dates, including periods of recruitment, exposure, follow-up, and data collection | | See “Methods” |
| Participants | 6 | (*a*) *Cohort study*—Give the eligibility criteria, and the sources and methods of selection of participants. Describe methods of follow-up  *Case-control study*—Give the eligibility criteria, and the sources and methods of case ascertainment and control selection. Give the rationale for the choice of cases and controls  *Cross-sectional study*—Give the eligibility criteria, and the sources and methods of selection of participants | | See “Methods” |
|  |  | (*b*) *Cohort study*—For matched studies, give matching criteria and number of exposed and unexposed  *Case-control study*—For matched studies, give matching criteria and the number of controls per case | | See “Study design and dnalysis” |
| Variables | 7 | Clearly define all outcomes, exposures, predictors, potential confounders, and effect modifiers. Give diagnostic criteria, if applicable | | See “Methods” and Appendices |
| Data sources/ measurement | 8* | For each variable of interest, give sources of data and details of methods of assessment (measurement). Describe comparability of assessment methods if there is more than one group | | See “Methods” |
| Bias | 9 | Describe any efforts to address potential sources of bias | | We adjusted the serum-creatinine values obtained before 2004 (when a different method was used). See “Measurements” |
| Study size | 10 | Explain how the study size was arrived at | | Not applicable. |

| Quantitative variables | 11 | Explain how quantitative variables were handled in the analyses. If applicable, describe which groupings were chosen and why | See “Study design and analysis” |
| --- | --- | --- | --- |
| Statistical methods | 12 | (*a*) Describe all statistical methods, including those used to control for confounding | See “Study design and analysis” |
|  |  | (*b*) Describe any methods used to examine subgroups and interactions | Subgroups could not be examined, due to insufficient size. |
|  |  | (*c*) Explain how missing data were addressed | We employed a statistical method that deals with loss of follow-up (Cumulative Incidence Function). We did not have missing data. |
|  |  | (*d*) *Cohort study*—If applicable, explain how loss to follow-up was addressed  *Case-control study*—If applicable, explain how matching of cases and controls was addressed  *Cross-sectional study*—If applicable, describe analytical methods taking account of sampling strategy | The statistical method employed deals with loss of follow-up. Matching for case-control study is explained in section “Study Design and Analysis”, see also Figure 2 |
|  |  | (*e*) Describe any sensitivity analyses | Not applicable |
| Participants | 13* | (a) Report numbers of individuals at each stage of study—eg numbers potentially eligible, examined for eligibility, confirmed eligible, included in the study, completing follow-up, and analysed | See “Results”, Figure 3 – Study Flow Chart. |
|  |  | (b) Give reasons for non-participation at each stage | See “Results”, Figure 3 – Study Flow Chart. |
|  |  | (c) Consider use of a flow diagram | See “Results”, Figure 3 – Study Flow Chart. |
| Descriptive data | 14* | (a) Give characteristics of study participants (eg demographic, clinical, social) and information on exposures and potential confounders | See “Results” |
|  |  | (b) Indicate number of participants with missing data for each variable of interest | Not applicable |
|  |  | (c) *Cohort study*—Summarise follow-up time (eg, average and total amount) | See Results. |
| Outcome data | 15* | *Cohort study*—Report numbers of outcome events or summary measures over time | See Results |
|  |  | *Case-control study—*Report numbers in each exposure category, or summary measures of exposure | See Results |
|  |  | *Cross-sectional study—*Report numbers of outcome events or summary measures | Not applicable |
| Main results | 16 | (*a*) Give unadjusted estimates and, if applicable, confounder-adjusted estimates and their precision (eg, 95% confidence interval). Make clear which confounders were adjusted for and why they were included | See Results |
|  |  | (*b*) Report category boundaries when continuous variables were categorized | See Results |
|  |  | (*c*) If relevant, consider translating estimates of relative risk into absolute risk for a meaningful time period | See Results |

Continued on next page

| Other analyses | 17 | Report other analyses done—eg analyses of subgroups and interactions, and sensitivity analyses | See Strengths and Limitations, and Appendix 8 |
| --- | --- | --- | --- |
| Key results | 18 | Summarise key results with reference to study objectives | See Conclusions |
| Limitations | 19 | Discuss limitations of the study, taking into account sources of potential bias or imprecision. Discuss both direction and magnitude of any potential bias | See Strengths and Limitations |
| Interpretation | 20 | Give a cautious overall interpretation of results considering objectives, limitations, multiplicity of analyses, results from similar studies, and other relevant evidence | See Discussion and Conclusion |
| Generalisability | 21 | Discuss the generalisability (external validity) of the study results | See Strengths and Limitations |
| Other information | |  |  |
| Funding | 22 | Give the source of funding and the role of the funders for the present study and, if applicable, for the original study on which the present article is based | See Funding |

*Give information separately for cases and controls in case-control studies and, if applicable, for exposed and unexposed groups in cohort and cross-sectional studies.

**Note:** An Explanation and Elaboration article discusses each checklist item and gives methodological background and published examples of transparent reporting. The STROBE checklist is best used in conjunction with this article (freely available on the Web sites of PLoS Medicine at http://www.plosmedicine.org/, Annals of Internal Medicine at http://www.annals.org/, and Epidemiology at http://www.epidem.com/). Information on the STROBE Initiative is available at www.strobe-statement.org.
