# Supplementary material for: Lifetime risk of severe kidney disease in lithium-treated patients: a retrospective study
Source: Int J Bipolar Disord. 2023 Dec 9;11:39. doi: 10.1186/s40345-023-00319-2 (PMC10710395; doi:10.1186/s40345-023-00319-2)
Supplement: Supplementary file 2 — Additional file 2: Appendix S2. List of abbreviations. Appendix S3. Example of computing Time on Li and Mean S-Li, using Area under the curve (AUC). Appendix S4. Identification of competing deaths. Appendix S5. Descriptive statistics of the matching variables for the case-control study. Appendix S6. Rationale for choosing Time on Li as measure for lithium exposure. Appendix S7. Results of logistic regression in the chosen model, including the matching variables. Appendix S8. Results of logistic regression for alternative categorisations of Time on Li. [file 40345_2023_319_MOESM2_ESM.docx]

**List of Appendices (2-7)**

[Appendix 2. List of abbreviations 2](#_Toc152162369)

[Appendix 3. Example of computing Time on Li and Mean S-Li, using Area under the curve (AUC) 3](#_Toc152162370)

[Appendix 4. Identification of competing deaths 4](#_Toc152162371)

[Appendix 5. Descriptive statistics of the matching variables for the case-control study 5](#_Toc152162372)

[Appendix 6. Rationale for choosing Time on Li as measure for lithium exposure 6](#_Toc152162373)

[Appendix 7. Results of logistic regression in the chosen model, including the matching variables 7](#_Toc152162374)

[Appendix 8. Results of logistic regression for alternative categorisations of Time on Li 9](#_Toc152162375)

[Alternative 1, 5 categories: Time on Li: 0 (≥1, <5 years), 1 (≥5, <10 years), 2 (≥10, <15 years), 3 (≥15, <20 years) and 4 (≥20 years) 9](#_Toc152162376)

[Alternative 2, 4 categories: Time on Li: 0 (≥1, <5 years), 1 (≥5, <10 years), 2 (≥10, <15 years) and 3 (≥15 years) 11](#_Toc152162377)

[Alternative 3, 4 categories: Time on Li: 0 (≥1, <5 years), 1 (≥5, <10 years), 2 (≥10, <20 years) and 3 (≥20 years) 13](#_Toc152162378)

[Alternative 4, 3 categories: Time on Li: 0 (≥1, <5 years), 1 (≥5, <15 years) and 2 (≥15 years) 15](#_Toc152162379)

[Alternative 5: Time on Li as a continuous variable 17](#_Toc152162380)

# Appendix 2. List of abbreviations

| CIF | Cumulative Incidence Function |
| --- | --- |
| CIF CKD4+ | Cumulative Incidence Function for Incident CKD4+, considering the competing risk of death |
| CKD | Chronic Kidney Disease |
| CKD4 | Chronic Kidney Disease grade 4 |
| CKD4+ | Chronic Kidney Disease grade 4 or higher |
| Comp. Deaths | Number of competing deaths / Cumulative Incidence of competing deaths |
| eGFR | Estimated Glomerular Filtration Rate |
| ESRD | End Stage Renal Disease |
| HR | Hazard Ratio |
| KM CKD4+ | Inverse of Kaplan Meier curve for Incident CKD4+ |
| OR | Odds Ratio |
| RR | Risk Ratio |
| RRT | Renal Replacement Therapy |
| S-Creatinine | Serum creatinine concentration |
| S-Li | Serum lithium concentration |
| SD | Standard Deviation |

# Appendix 3. Example of computing Time on Li and Mean S-Li, using Area under the curve (AUC)

Figure 1 below is a diagram of the S-Li measurements for a fictive patient.


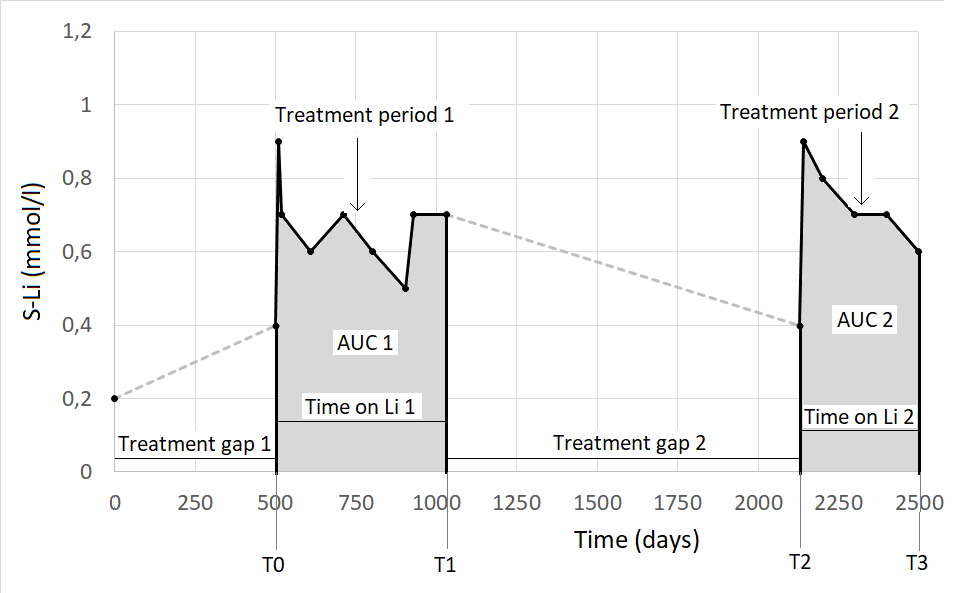


Figure 1 Diagram of S-Li values for a fictive patient. The dots represent S-Li measurements. The horizontal axis represents the time in days from the first S-Li measurement, the vertical axis represents the value for S-Li measurements.

Two treatment gaps (more than 1 years without a S-Li) and two treatment periods (greyed) can be observed in this fictional diagram.

The first treatment period starts at day 500 (T0) and ends at day 100 (T1). Therefore, T0 marks the start of Index Treatment and the beginning of follow up. The second treatment period starts at day 2130 (T2) and ends at day 2500 (T3).

Each treatment period is characterised by a Time on Li and an Area under the curve (AUC) – the grey areas in the diagram.

For every treatment period, we can compute Mean S-Li as the AUC / Time on Li.

For the total time interval presented in the diagram:
 Time on Li = Time on Li 1 + Time on Li 2
 Mean S-Li = (AUC 1 + AUC 2) / (Time on Li 1 + Time on Li 2)

**Generally, for a given patient and a given time interval that includes n treatment periods:
 Time on Li = sum of Time on Li (for all treatment periods, from 1 to n)
 Mean S-Li = sum of AUC (from 1 to n) / sum of Time on Li (from 1 to n).**

# Appendix 4. Identification of competing deaths

Competing Death status was assigned automatically to:

- Individuals who died during the study and had their Last eGFR of 40 ml/min/1.73 m² or more within their last 3 months of life (thus ruling out Incident CKD4+ before death). They were observed until date of death.

Censored status was assigned automatically to:

- Individuals alive at the end of the study (December 31. 2017). They were censored at the date of their Last creatinine.
- Individuals who died during the study but lacked S-creatinine values during the last 3 months of life (and thus had an unknown CKD4+ status at death). They were censored at the date of Last creatinine.

Following were manually examined by one of the authors:

- Individuals who died during the study with Last eGFR below 40 ml/min/1.73 m² and within the last 3 months. After examination, they were treated as follows:
  - If their S-creatinine/eGFR data allowed to rule out Incident CKD4+ before death. they were regarded as competing deaths and were observed until date of death.
  - If their S-creatinine/eGFR data did not allow to rule out Incident CKD4+ before death. they were censored at their Last creatinine.

# Appendix 5. Descriptive statistics of the matching variables for the case-control study

|  |  | Cases (N=103) | | Controls (N=412) | | p |
| --- | --- | --- | --- | --- | --- | --- |
| Variable | Category | Frequency | Percent | Frequency | Percent |  |
| Sex |  |  |  |  |  | 1 |
|  | 0 (men) | 32 | 31.1 | 128 | 31.1 | ^a^ |
|  | 1 (women) | 71 | 68.9 | 284 | 68.9 | ^a^ |
| Matched age |  |  |  |  |  | 1 |
|  | 0 (40-59 years) | 9 | 8.7 | 36 | 8.7 | ^a^ |
|  | 1 (60-69 years) | 29 | 28.2 | 116 | 28.2 | ^a^ |
|  | 2 (70-79 years) | 40 | 38.8 | 160 | 38.8 | ^a^ |
|  | 3 (≥80 years) | 25 | 24.3 | 100 | 24.3 | ^a^ |

^a^ Proportions are identical.

# Appendix 6. Rationale for choosing Time on Li as measure for lithium exposure

The total lithium exposure is mathematically best described by the Area under the Curve (*AUC*) which takes into consideration both the duration of treatment (*Time on Li*) and the *Mean S-Li.*

In our patient population, *Time on Li* and *AUC* were highly correlated. This was valid for the population included both in the cohort study and in the case-control study. (see Figure 1a and b)


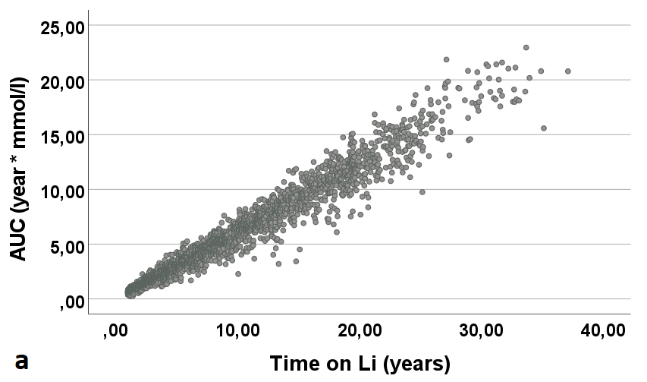

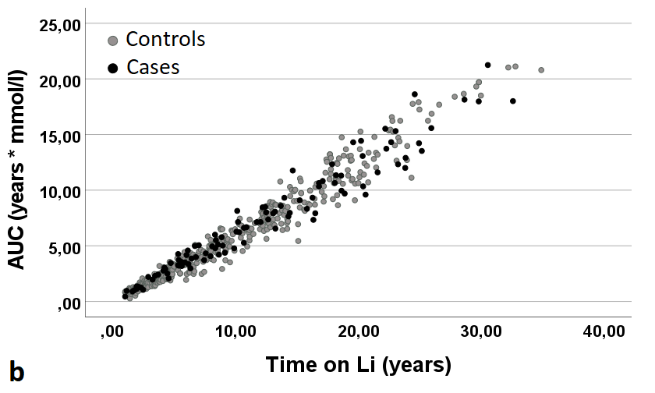


Fig 2. Scatterplot AUC vs Time on Li for: a) the whole cohort (N=2381 patients) and b) the case-control population. (N=515 patients)

Pearson correlation coefficient for the whole cohort was 0.978 (95%CI 0.976-0.980) and for the case-control population 0.977 (95% CI 0.973-0.981).

This strong correlation is explained by the tightly controlled *Mean S-Li* levels, with small inter- individual variations. *Mean S-Li* was: 0.60 (SD 0.10) mmol/L for the whole cohort, 0.59 (SD 0.10) mmol/l for Controls and 0.61 (SD 0.09) mmol/l for Cases (See Fig 2a and b).


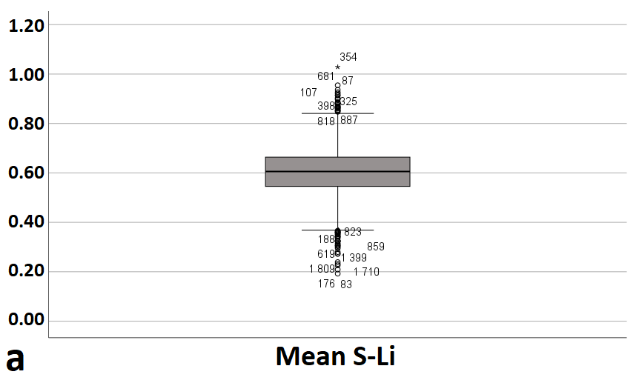

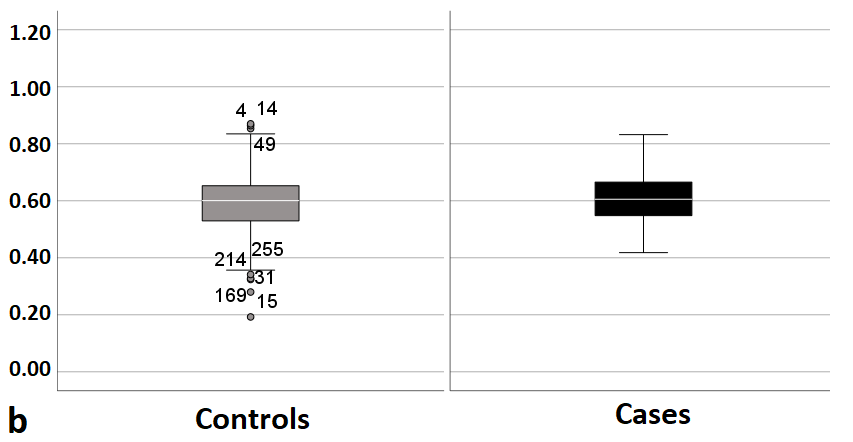


Fig 2. Boxplot of Mean S-Li for: a) the whole cohort (N=2381 patients) and b) the case-control population (Controls = 412, Cases = 103, Total = 515 patients)

***Time on Li* was chosen as a proxy measure of lithium exposure (rather than *AUC*) due to its simplicity and readily availability for both physicians and patients.**

# Appendix 7. Results of logistic regression in the chosen model, including the matching variables

Fully adjusted model (adjusted for matching variables and Start creatinine)

|  |  |  |  | 95% C.I.for OR | |  |
| --- | --- | --- | --- | --- | --- | --- |
| Variable | Category | Reference | OR | Lower | Upper | p-value |
| Time on Li |  |  |  |  |  | <0.001 |
|  | 0 (1≤ x <5 years) | REF |  |  |  |  |
|  | 1 (5≤ x <20 years) |  | 2.29 | 1.25 | 4.19 | 0.007 |
|  | 2 (x ≥20 years) |  | 5.85 | 2.54 | 13.44 | <0.001 |
| Start creatinine |  |  |  |  |  | <0.001 |
|  | 0 (lower third) | REF |  |  |  |  |
|  | 1 (middle third) |  | 2.15 | 1.27 | 3.64 | 0.004 |
|  | 2 (upper third) |  | 6.84 | 3.55 | 13.18 | <0.001 |
| Sex |  |  |  |  |  |  |
|  | 0 ( men) | REF |  |  |  |  |
|  | 1 (women) |  | 0.98 | 0.59 | 1.62 | 0.932 |
| Matching Year |  |  | 0.99 | 0.95 | 1.02 | 0.470 |
| Matched age |  |  |  |  |  | 0.985 |
|  | 0 (40-59 years) | REF |  |  |  |  |
|  | 1 (60-69 years) |  | 0.89 | 0.36 | 2.19 | 0.798 |
|  | 2 (70-79 years) |  | 0.97 | 0.41 | 2.27 | 0.933 |
|  | 3 (≥ 80 years) |  | 1.00 | 0.40 | 2.46 | 0.995 |

OR = Odds Ratio. 95%CI = 95% Confidence Interval. REF = the reference category in each categorical variable.
Number of patients included in the model: 515. Outcome variable: Incident CKD4+. Predictor variable: Time on Li (categorised), adjusted for Start Creatinine (categorised) and matching variables Sex, Matching year and Matched age group).
Predictive efficiency: 79.8% (cut-off value 0.5). Hosmer-Lemeshow test p=0.799.

Partly adjusted model (adjusted for matching variables only):

|  |  |  |  | 95% C.I.for OR | |  |
| --- | --- | --- | --- | --- | --- | --- |
| Variable | Category | Reference | OR | Lower | Upper | p-value |
| Time on Li |  |  |  |  |  | 0.006 |
|  | 0 (1≤ x <5 years) | REF |  |  |  |  |
|  | 1 (5≤ x <20 years) |  | 1.80 | 1.003 | 3.13 | 0.049 |
|  | 2 (x ≥20 years) |  | 3.52 | 1.62 | 7.65 | 0.001 |
| Sex |  |  |  |  |  | 0.881 |
|  | 0 ( men) | REF |  |  |  |  |
|  | 1 (women) |  | 0,96 | 0.60 | 1,56 | 0.881 |
| Matching Year |  |  | 0.98 | 0.95 | 1.02 | 0.286 |
| Matched age |  |  |  |  |  | 1.00 |
|  | 0 (40-59 years) | REF |  |  |  |  |
|  | 1 (60-69 years) |  | 0.95 | 0.40 | 2.26 | 0.91 |
|  | 2 (70-79 years) |  | 0.94 | 0.41 | 2.14 | 0.89 |
|  | 3 (≥ 80 years) |  | 1.00 | 0.42 | 2.36 | 0.99 |

Unadjusted model:

|  |  |  |  | 95% C.I.for OR | |  |
| --- | --- | --- | --- | --- | --- | --- |
| Variable | Category | Reference | OR | Lower | Upper | p-value |
| Time on Li |  |  |  |  |  | 0,011 |
|  | 0 (1≤ x <5 years) | REF |  |  |  |  |
|  | 1 (5≤ x <20 years) |  | 1,66 | 0,95 | 2,90 | 0,073 |
|  | 2 (x ≥20 years) |  | 2,99 | 1,46 | 6,12 | 0,003 |

# Appendix 8. Results of logistic regression for alternative categorisations of Time on Li

## Alternative 1, 5 categories: Time on Li: 0 (≥1, <5 years), 1 (≥5, <10 years), 2 (≥10, <15 years), 3 (≥15, <20 years) and 4 (≥20 years)

Fully adjusted model (adjusted for matching variables and Start creatinine)

|  |  |  |  | 95%CI for OR | |  |
| --- | --- | --- | --- | --- | --- | --- |
| Variable | Category | Reference | OR | Lower | Upper | p-value |
| Time on Li |  |  |  |  |  | 0.001 |
|  | 0 (1≤ x <5 years) | REF |  |  |  |  |
|  | 1 (5≤ x <10 years) |  | 2.04 | 1.04 | 4.00 | 0.039 |
|  | 2 (10≤ x <15 years) |  | 2.40 | 1.15 | 5.03 | 0.020 |
|  | 3 (15≤ x <20 years) |  | 2.94 | 1.27 | 6.82 | 0.012 |
|  | 4 (x ≥20 years) |  | 6.14 | 2.65 | 14.27 | <0.001 |
| Start Creatinine |  |  |  |  |  | <0.001 |
|  | 0 (lower third) | REF |  |  |  |  |
|  | 1 (middle third) |  | 2.23 | 1.31 | 3.82 | 0.003 |
|  | 2 (upper third) |  | 7.00 | 3.62 | 13.54 | <0.001 |
| Sex |  |  |  |  |  | 0.902 |
|  | 0 (men) | REF |  |  |  |  |
|  | 1 (women) |  | 0.97 | 0.59 | 1.60 | 0.902 |
| Matching Year |  |  | 0.98 | 0.95 | 1.02 | 0.342 |
| Matched Age |  |  |  |  |  | 0.987 |
|  | 0 (40-59 years) | REF |  |  |  |  |
|  | 1 (60-69 years) |  | 0.90 | 0.37 | 2.22 | 0.821 |
|  | 2 (70-79 years) |  | 0.96 | 0.41 | 2.25 | 0.916 |
|  | 3 (≥ 80 years) |  | 1.01 | 0.41 | 2.50 | 0.982 |

OR = Odds Ratio. 95%CI = 95% Confidence Interval. REF = the reference category in each categorical variable.
Number of patients included in the model: 515. Outcome variable: Incident CKD4+. Predictor variable: Time on Li (categorised). Adjusted for Start Creatinine (categorised) and matching variables Sex. Matching year and Matched age group.
Predictive efficiency: 79.4% (cut-off value 0.5). Hosmer-Lemeshow test p=0.306.

Partly adjusted model (adjusted for matching variables only):

|  |  |  |  | 95% C.I.for OR | |  |
| --- | --- | --- | --- | --- | --- | --- |
| Variable | Category | Reference | OR | Lower | Upper | p-value |
| Time on Li |  |  |  |  |  | 0.036 |
|  | 0 (1≤ x <5 years) | REF |  |  |  |  |
|  | 1 (5≤ x <10 years) |  | 1.67 | 0.88 | 3.16 | 0.119 |
|  | 2 (10≤ x <15 years) |  | 1.85 | 0.92 | 3.71 | 0.085 |
|  | 3 (15≤ x <20 years) |  | 1.94 | 0.89 | 4.25 | 0.097 |
|  | 4 (x ≥20 years) |  | 3.58 | 1.64 | 7.82 | 0.001 |
| Sex |  |  |  |  |  | 0.879 |
|  | 0 ( men) | REF |  |  |  |  |
|  | 1 (women) |  | 0.96 | 0.60 | 1.56 | 0.879 |
| Matching Year |  |  | 0.98 | 0.95 | 1.02 | 0.255 |
| Matched age |  |  |  |  |  | 0.997 |
|  | 0 (40-59 years) | REF |  |  |  |  |
|  | 1 (60-69 years) |  | 0.95 | 0.40 | 2.27 | 0.913 |
|  | 2 (70-79 years) |  | 0.94 | 0.41 | 2.13 | 0.872 |
|  | 3 (≥ 80 years) |  | 0.99 | 0.42 | 2.36 | 0.984 |

Unadjusted model

|  |  |  |  | 95%CI for OR | |  |
| --- | --- | --- | --- | --- | --- | --- |
| Variable | Category | Reference | OR | Lower | Upper | p-value |
| Time on Li |  |  |  |  |  | 0.062 |
|  | 0 (1≤ x <5 years) | REF |  |  |  |  |
|  | 1 (5≤ x <10 years) |  | 1.62 | 0.86 | 3.06 | 0.138 |
|  | 2 (10≤ x <15 years) |  | 1.71 | 0.86 | 3.38 | 0.124 |
|  | 3 (15≤ x <20 years) |  | 1.69 | 0.80 | 3.56 | 0.168 |
|  | 4 (x ≥20 years) |  | 2.99 | 1.46 | 6.12 | 0.003 |

## Alternative 2, 4 categories: Time on Li: 0 (≥1, <5 years), 1 (≥5, <10 years), 2 (≥10, <15 years) and 3 (≥15 years)

Fully adjusted model (adjusted for matching variables and Start creatinine)

|  |  |  |  | 95%CI for OR | |  |
| --- | --- | --- | --- | --- | --- | --- |
| Variable | Category | Reference | OR | Lower | Upper | p-value |
| Time on Li |  |  |  |  |  | 0.02 |
|  | 0 (1≤ x <5 years) | REF |  |  |  |  |
|  | 1 (5≤ x <10 years) |  | 2.02 | 1.03 | 3.96 | 0.041 |
|  | 2 (10≤ x <15 years) |  | 2.36 | 1.13 | 4.92 | 0.023 |
|  | 3 (x ≥15 years) |  | 4.15 | 2.00 | 8.59 | <0.001 |
| Start Creatinine |  |  |  |  |  | <0.001 |
|  | 0 (lower third) | REF |  |  |  |  |
|  | 1 (middle third) |  | 2.24 | 1.31 | 3.83 | 0.003 |
|  | 2 (upper third) |  | 6.76 | 3.51 | 13.03 | <0.001 |
| Sex |  |  |  |  |  | 0.961 |
|  | 0 (men) | REF |  |  |  |  |
|  | 1 (women) |  | 0.99 | 0.60 | 1.63 | 0.961 |
| Matching Year |  |  | 0.98 | 0.95 | 1.02 | 0.391 |
| Matched Age |  |  |  |  |  | 0.991 |
|  | 0 (40-59 years) | REF |  |  |  |  |
|  | 1 (60-69 years) |  | 0.93 | 0.38 | 2.29 | 0.873 |
|  | 2 (70-79 years) |  | 0.98 | 0.42 | 2.29 | 0.955 |
|  | 3 (≥ 80 years) |  | 1.034 | 0.42 | 2.55 | 0.943 |

OR = Odds Ratio. 95%CI = 95% Confidence Interval. REF = the reference category in each categorical variable.
Number of patients included in the model: 515. Outcome variable: Incident CKD4+. Predictor variable: Time on Li (categorised). adjusted for Start Creatinine (categorised) and matching variables Sex. Matching year and Matched age group.
Predictive efficiency: 79.0% (cut-off value 0.5). Hosmer-Lemeshow test p=0.382.

Partly adjusted model (adjusted for matching variables only):

|  |  |  |  | 95% C.I.for OR | |  |
| --- | --- | --- | --- | --- | --- | --- |
| Variable | Category | Reference | OR | Lower | Upper | p-value |
| Time on Li |  |  |  |  |  | 0.053 |
|  | 0 (1≤ x <5 years) | REF |  |  |  |  |
|  | 1 (5≤ x <10 years) |  | 1.66 | 0.88 | 3.15 | 0.121 |
|  | 2 (10≤ x <15 years) |  | 1.83 | 0.91 | 03.67 | 0.089 |
|  | 3 (x ≥15 years) |  | 2.59 | 1.32 | 5.06 | 0.006 |
| Sex |  |  |  |  |  | 0.948 |
|  | 0 ( men) | REF |  |  |  |  |
|  | 1 (women) |  | 0.98 | 0.61 | 1.59 | 0.948 |
| Matching Year |  |  | 0.98 | 0.95 | 1.02 | 0.309 |
| Matched age |  |  |  |  |  | 0.998 |
|  | 0 (40-59 years) | REF |  |  |  |  |
|  | 1 (60-69 years) |  | 0.97 | 0.41 | 2.30 | 0.944 |
|  | 2 (70-79 years) |  | 0.95 | 0.42 | 2.16 | 0.905 |
|  | 3 (≥ 80 years) |  | 1.00 | 0.42 | 2.39 | 0.992 |

Unadjusted model

|  |  |  |  | 95%CI for OR | |  |
| --- | --- | --- | --- | --- | --- | --- |
| Variable | Category | Reference | OR | Lower | Upper | p-value |
| Time on Li |  |  |  |  |  | 0.085 |
|  | 0 (1≤ x <5 years) | REF |  |  |  |  |
|  | 1 (5≤ x <10 years) |  | 1.62 | 0.86 | 3.06 | 0.138 |
|  | 2 (10≤ x <15 years) |  | 1.71 | 0.86 | 3.38 | 0.124 |
|  | 3 (x ≥15 years) |  | 2.25 | 1.21 | 4.17 | 0.010 |

## Alternative 3, 4 categories: Time on Li: 0 (≥1, <5 years), 1 (≥5, <10 years), 2 (≥10, <20 years) and 3 (≥20 years)

Fully adjusted model (adjusted for matching variables and Start creatinine)

|  |  |  |  | 95%CI for OR | |  |
| --- | --- | --- | --- | --- | --- | --- |
| Variable | Category | Reference | OR | Lower | Upper | p-value |
| Time on Li |  |  |  |  |  | <0.001 |
|  | 0 (1≤ x <5 years) | REF |  |  |  |  |
|  | 1 (5≤ x <10 years) |  | 2.04 | 1.04 | 4.00 | 0.039 |
|  | 2 (10≤ x <20 years) |  | 2.59 | 1.32 | 5.07 | 0.006 |
|  | 3 (x ≥20 years) |  | 6.04 | 2.61 | 13.98 | <0.001 |
| Start Creatinine |  |  |  |  |  | <0.001 |
|  | 0 (lower third) | REF |  |  |  |  |
|  | 1 (middle third) |  | 2.17 | 1.29 | 3.67 | 0.004 |
|  | 2 (upper third) |  | 6.96 | 3.60 | 13.45 | <0.001 |
| Sex |  |  |  |  |  | 0.923 |
|  | 0 (men) | REF |  |  |  |  |
|  | 1 (women) |  | 0.98 | 0.60 | 1.61 | 0.923 |
| Matching Year |  |  | 0.98 | 0.95 | 1.02 | 0.381 |
| Matched Age |  |  |  |  |  | 0.988 |
|  | 0 (40-59 years) | REF |  |  |  |  |
|  | 1 (60-69 years) |  | 0.90 | 0.36 | 2.20 | 0.806 |
|  | 2 (70-79 years) |  | 0.96 | 0.41 | 2.25 | 0.917 |
|  | 3 (≥ 80 years) |  | 1.00 | 0.40 | 2.46 | 0.994 |

OR = Odds Ratio. 95%CI = 95% Confidence Interval. REF = the reference category in each categorical variable.
Number of patients included in the model: 515. Outcome variable: Incident CKD4+. Predictor variable: Time on Li (categorised). adjusted for Start Creatinine (categorised) and matching variables Sex. Matching year and Matched age group.
Predictive efficiency: 79.8% (cut-off value 0.5). Hosmer-Lemeshow test p=0.195.

Partly adjusted model (adjusted for matching variables only):

|  |  |  |  | 95% C.I.for OR | |  |
| --- | --- | --- | --- | --- | --- | --- |
| Variable | Category | Reference | OR | Lower | Upper | p-value |
| Time on Li |  |  |  |  |  | 0.017 |
|  | 0 (1≤ x <5 years) | REF |  |  |  |  |
|  | 1 (5≤ x <10 years) |  | 1.67 | 0.88 | 3.16 | 0.119 |
|  | 2 (10≤ x <20 years) |  | 1.89 | 0.999 | 3.54 | 0.050 |
|  | 3 (x ≥20 years) |  | 3.57 | 1.64 | 7.79 | 0.001 |
| Sex |  |  |  |  |  | 0.881 |
|  | 0 ( men) | REF |  |  |  |  |
|  | 1 (women) |  | 0.96 | 0.60 | 1.56 | 0.881 |
| Matching Year |  |  | 0.98 | 0.95 | 1.02 | 0.258 |
| Matched age |  |  |  |  |  | 0.997 |
|  | 0 (40-59 years) | REF |  |  |  |  |
|  | 1 (60-69 years) |  | 0.95 | 0.40 | 2.26 | 0.909 |
|  | 2 (70-79 years) |  | 0.94 | 0.41 | 2.13 | 0.872 |
|  | 3 (≥ 80 years) |  | 0.99 | 0.42 | 2.35 | 0.978 |

Unadjusted model

|  |  |  |  | 95%CI for OR | |  |
| --- | --- | --- | --- | --- | --- | --- |
| Variable | Category | Reference | OR | Lower | Upper | p-value |
| Time on Li |  |  |  |  |  | 0.030 |
|  | 0 (1≤ x <5 years) | REF |  |  |  |  |
|  | 1 (5≤ x <10 years) |  | 1.62 | 0.86 | 3.06 | 0.138 |
|  | 2 (10≤ x <20 years) |  | 1.70 | 0.93 | 3.12 | 0.087 |
|  | 3 (x ≥20 years) |  | 2.99 | 1.46 | 6.12 | 0.003 |

## Alternative 4, 3 categories: Time on Li: 0 (≥1, <5 years), 1 (≥5, <15 years) and 2 (≥15 years)

Fully adjusted model (adjusted for matching variables and Start creatinine)

|  |  |  |  | 95% C.I.for OR | |  |
| --- | --- | --- | --- | --- | --- | --- |
| Variable | Category | Reference | OR | Lower | Upper | p-value |
| Time on Li |  |  |  |  |  | <0.001 |
|  | 0 (1≤ x <5 years) | REF |  |  |  |  |
|  | 1 (5≤ x <15 years) |  | 2.15 | 1.16 | 3.98 | 0.015 |
|  | 2 (x ≥15 years) |  | 4.11 | 1.99 | 8.50 | <0.001 |
| Start creatinine |  |  |  |  |  | <0.001 |
|  | 0 (lower third) | REF |  |  |  |  |
|  | 1 (middle third) |  | 2.26 | 1.32 | 3.85 | 0.003 |
|  | 2 (upper third) |  | 6.71 | 3.49 | 12.92 | <0.001 |
| Sex |  |  |  |  |  | 0.957 |
|  | 0 ( men) | REF |  |  |  |  |
|  | 1 (women) |  | 0.99 | 0.60 | 1.63 | 0.957 |
| Matching Year |  |  | 0.99 | 0.95 | 1.02 | 0.416 |
| Matched age |  |  |  |  |  | 0.989 |
|  | 0 (40-59 years) | REF |  |  |  |  |
|  | 1 (60-69 years) |  | 0.93 | 0.38 | 2.29 | 0.874 |
|  | 2 (70-79 years) |  | 0.98 | 0.42 | 2.30 | 0.962 |
|  | 3 (≥ 80 years) |  | 1.04 | 0.42 | 2.56 | 0.933 |

OR = Odds Ratio. 95%CI = 95% Confidence Interval. REF = the reference category in each categorical variable.
Number of patients included in the model: 515. Outcome variable: Incident CKD4+. Predictor variable: Time on Li (categorised). adjusted for Start Creatinine (categorised) and matching variables Sex. Matching year and Matched age group.
Predictive efficiency: 79.4% (cut-off value 0.5). Hosmer-Lemeshow test p=0.076.

Partly adjusted model (adjusted for matching variables only):

|  |  |  |  | 95% C.I.for OR | |  |
| --- | --- | --- | --- | --- | --- | --- |
| Variable | Category | Reference | OR | Lower | Upper | p-value |
| Time on Li |  |  |  |  |  | 0.022 |
|  | 0 (1≤ x <5 years) | REF |  |  |  |  |
|  | 1 (5≤ x <15 years) |  | 1.73 | 0.96 | 3.10 | 0.067 |
|  | 2 (x ≥15 years) |  | 2.57 | 1.31 | 5.03 | 0.006 |
| Sex |  |  |  |  |  | 0.945 |
|  | 0 ( men) | REF |  |  |  |  |
|  | 1 (women) |  | 0.98 | 0.61 | 1.59 | 0.945 |
| Matching Year |  |  | 0.98 | 0.95 | 1.02 | 0.323 |
| Matched age |  |  |  |  |  | 0.998 |
|  | 0 (40-59 years) | REF |  |  |  |  |
|  | 1 (60-69 years) |  | 0.97 | 0.41 | 2.31 | 0.949 |
|  | 2 (70-79 years) |  | 0.96 | 0.42 | 2.17 | 0.913 |
|  | 3 (≥ 80 years) |  | 1.01 | 0.43 | 2.40 | 0.983 |

Unadjusted model

|  |  |  |  | 95% C.I.for OR | |  |
| --- | --- | --- | --- | --- | --- | --- |
| Variable | Category | Reference | OR | Lower | Upper | p-value |
| Time on Li |  |  |  |  |  | 0.037 |
|  | 0 (1≤ x <5 years) | REF |  |  |  |  |
|  | 1 (5≤ x <15 years) |  | 1.66 | 0.93 | 2.95 | 0.086 |
|  | 2 (x ≥15 years) |  | 2.25 | 1.21 | 4.17 | 0.010 |

## Alternative 5: Time on Li as a continuous variable

Fully adjusted model (adjusted for matching variables and Start creatinine)

|  |  |  |  | 95% CI for OR | |  |
| --- | --- | --- | --- | --- | --- | --- |
| Variable | Category | Reference | OR | Lower | Upper | p-value |
| Time on Li (scaled 5:1)* |  |  | 1.40 | 1.17 | 1.66 | <0.001 |
| Start Creatinine |  |  |  |  |  | <0.001 |
|  | 0 (lower third) | REF |  |  |  |  |
|  | 1 (middle third) |  | 2.25 | 1.33 | 3.80 | 0.003 |
|  | 2 (upper third) |  | 6.50 | 3.40 | 12.43 | <0.001 |
| Sex |  |  |  |  |  | 0.853 |
|  | 0 (men) | REF |  |  |  |  |
|  | 1 (women) |  | 0.95 | 0.58 | 1.57 | 0.853 |
| Matching Year |  |  | 0.98 | 0.95 | 1.02 | 0.338 |
| Matched Age |  |  |  |  |  | 0.997 |
|  | 0 (40-59 years) | REF |  |  |  |  |
|  | 1 (60-69 years) |  | 0.92 | 0.38 | 2.25 | 0.854 |
|  | 2 (70-79 years) |  | 0.95 | 0.41 | 2.22 | 0.903 |
|  | 3 (≥ 80 years) |  | 0.98 | 0.40 | 2.40 | 0.958 |

OR = Odds Ratio. 95%CI = 95% Confidence Interval. REF = the reference category in each categorical variable.
Number of patients included in the model: 515. Outcome variable: Incident CKD4+. Predictor variable: Time on li. Model adjusted for Start Creatinine (categorised) and matching variables (Sex. Matching year and Matched age group).
Predictive efficiency: 79.2% (cut-off value 0.5). Hosmer-Lemeshow test p=0.916.

*Time on Li (continuous) is scaled 5:1. i.e., every unit of the scaled Time on Li represents 5 calendar years.

Partly adjusted model (adjusted for matching variables only):

|  |  |  |  | 95% C.I.for OR | |  |
| --- | --- | --- | --- | --- | --- | --- |
| Variable | Category | Reference | OR | Lower | Upper | p-value |
| Time on Li (scaled 5:1)* |  |  | 1.25 | 1.07 | 1.47 | 0.006 |
| Sex |  |  |  |  |  | 0.872 |
|  | 0 ( men) | REF |  |  |  |  |
|  | 1 (women) |  | 0.96 | 0.60 | 1.56 | 0.872 |
| Matching Year |  |  | 0.98 | 0.95 | 1.02 | 0.27 |
| Matched age |  |  |  |  |  | 0.998 |
|  | 0 (40-59 years) | REF |  |  |  |  |
|  | 1 (60-69 years) |  | 0.97 | 0.41 | 2.30 | 0.945 |
|  | 2 (70-79 years) |  | 0.94 | 0.41 | 2.13 | 0.877 |
|  | 3 (≥ 80 years) |  | 0.98 | 0.41 | 2.31 | 0.957 |

*Time on Li (continuous) is scaled 5:1. i.e., every unit of the scaled Time on Li represents 5 calendar years.

Unadjusted model

|  |  |  |  | 95% CI for OR | |  |
| --- | --- | --- | --- | --- | --- | --- |
| Variable | Category | Reference | OR | Lower | Upper | p-value |
| Time on Li (scaled 5:1)* |  |  | 1.20 | 1.04 | 1.39 | 0.012 |

*Time on Li (continuous) is scaled 5:1. i.e., every unit of the scaled Time on Li represents 5 calendar years.
